# WhatsApp-Delivered Intervention for Continued Learning for Nurses in Pakistan During the COVID-19 Pandemic: Results of a Randomized-Controlled Trial

**DOI:** 10.3389/fpubh.2022.739761

**Published:** 2022-02-15

**Authors:** Sara Rizvi Jafree, Rubeena Zakar, Nasim Rafiq, Ambreen Javed, Rana Rubab Durrani, Syeda Khadija Burhan, Syed Mujtaba Hasnain Nadir, Fatima Ali, Aimen Shahid, Ain ul Momina, Kamil J. Wrona, Qaisar Khalid Mahmood, Florian Fischer

**Affiliations:** ^1^Department of Sociology, Forman Christian College University, Lahore, Pakistan; ^2^Department of Public Health, Institute of Social and Cultural Studies, University of the Punjab, Lahore, Pakistan; ^3^Shalamar Nursing College, Lahore, Pakistan; ^4^Department of English, Forman Christian College University, Lahore, Pakistan; ^5^Language Development Center, Forman Christian College University, Lahore, Pakistan; ^6^Health Education England, North West Deanery, Leeds, United Kingdom; ^7^CMH Lahore Medical College, Lahore, Pakistan; ^8^Institute of Public Health, King Edward Medical University, Lahore, Pakistan; ^9^School of Public Health, Bielefeld University, Bielefeld, Germany; ^10^Department of Sociology, International Islamic University, Islamabad, Pakistan; ^11^Institute of Public Health, Charité – Universitätsmedizin Berlin, Berlin, Germany; ^12^Institute of Gerontological Health Services and Nursing Research, Ravensburg-Weingarten University of Applied Sciences, Weingarten, Germany; ^13^Bavarian Research Center for Digital Health and Social Care, Kempten University of Applied Sciences, Kempten, Germany

**Keywords:** nurse, online-based, digital, infection prevention, corona, coronavirus, SARS-CoV-2

## Abstract

The COVID-19 pandemic has necessitated support for continued learning in frontline practitioners through online digital mediums that are convenient and fast to maintain physical distancing. Nurses are already neglected professionals for support in training for infection control, leadership, and communication in Pakistan and other developing countries. For that reason, we aimed to deliver a WhatsApp-based intervention for continued learning in nurses who are currently working in both private and public sector. A 12-week intervention was delivered to 208 nurses (102 in the control group and 106 in the intervention group) who had been employed in the clinical setting during data collection. The analysis reveals that nurses in the intervention group show significantly better results for learning in “infection prevention and control” and “leadership and communication.” Results of a content analysis based on participant's feedback also confirm that the WhatsApp-based intervention is a valuable tool for education. This study highlights the effectiveness of online-based digital interventions as a convenient training tool for awareness and management of infectious diseases, leadership, and communication during COVID-19 and beyond. Furthermore, this study emphasizes that group interventions with other healthcare practitioners and the role of on-going longer WhatsApp-based interventions can become integral tools to support continued learning and patient safety practices.

## Introduction

Nurses across the globe have been struggling with multiple and compounding challenges since the onslaught of the COVID-19 pandemic (1). A range of challenges have been reported in the literature, including shortages of Personal Protective Equipment (PPE) (2), deficiencies in staffing and compensation (3), inability to maintain physical distancing due to nature of job (4), and fear of acquiring the infection and passing it to family members (5). However, less attention has been given to the predicament of continued learning during the pandemic (6). Continued learning in nurses is empirically associated with lower mortality and morbidity among patients (7). Empowering nurses with skill development and on-the-job training also has benefits for nurse's self-esteem and their job satisfaction (8). In addition, continued learning is known to provide nurses with better opportunities for professional development and career advancement (9, 10).

Nurses require support for continued learning in three keys areas during the pandemic. Firstly, learning for infection prevention and control is needed to prevent exposure to infection and patient placement and isolation precautions (11). Despite the fact that nurses are frontline practitioners and more frequently exposed to infectious disease in the clinical setting (12), there has been inadequate support for infection control training since the outbreak of the COVID-19 pandemic (13). The training that has been delivered has been criticized for being either too long and too difficult to follow, or too short with lack of opportunity for providing questions and receiving adequate answers (14).

Different learning elements related to infection prevention and control are associated with improved care delivery practices in nurses and recovery rates for patients. These learning elements include better understanding of infection prevention and control, improved planning and monitoring, and the ability to evaluate oneself and colleagues (15). With both the Ebola and COVID-19 pandemic, the lack of nurse preparation for infection prevention has contributed to a delayed response and increased rate of mortality and morbidity (16, 17). Coping capacities of nurses are linked to the combination of pre-service and on-the-job learning support which provide positive incentives for functional efficiency and patient safety culture.

Secondly, nurses are in need for continued learning about COVID-19 (18). Some literature confirms that less than half of healthcare practitioners, including nurses, have knowledge about COVID-19 and patient management (19). Other research suggests that the majority of nurses and other healthcare practitioners are dependent on social media for updates about disease management (20) and need regular and intensive learning support (21). Ongoing and updated knowledge of COVID-19 is integral for nurses, and other frontlines workers, during the pandemic as it shapes patient care protocols and successful discharge rates (22). Knowledge and continued learning of the management of COVID-19 patients is also important in emergency situations and during urgent care coordination between healthcare providers.

Better knowledge of disease epidemiology in nurses is positively associated with reduced adverse patient outcomes including misdiagnosis and medication errors (23). As highlighted by International Labor Organization and World Bank, the novelty of the disease and guidelines for the management protocols of mild to severe patients of COVID-19 is evolving and, thus, there is need for continued training for nurses and other healthcare practitioners (24). Effective learning support for disease knowledge and management can improve the overall performance capacity of healthcare service delivery, especially in resource short developing regions. Furthermore, it is a vital factor that improves job satisfaction and emotional health of nurses during critical times (25).

Thirdly, nurses need support in continued learning for leadership and communication skills (26). This is especially relevant during pandemics when nurses are required to play a more autonomous and self-reliant role in emergency situations (27). As frontline providers, each nurse, regardless of designation, must be supported for continued education to assume leadership roles. The ability of nurses to have skills for leadership, to speak up, and come up with innovative ideas in emergency situations is important (28). Nurses that are empowered with continued learning for leadership and management during the pandemic are in a better position to take responsibility for efficient care delivery to patients.

Healthcare providers, including nurses, are particularly vulnerable to communication problems in the current pandemic, given its unique and uncertain nature (29). Evidence highlights that the pandemic has left nurses with more burnout and stress due to the increase in communication problems and conflict at the workplace (30). This is primarily due to the difficult circumstances, but also because nurses bear the brunt of negative reactions in hospital settings and public zones due to lower professional status (31). It is important that nurses are able to manage conflict at the workplace for their emotional health, but also to secure quality services for patients and family attendants (32). Similarly, improved communication between nurses and colleagues is integral for preventing psychological distress in nurses and improving patient outcomes (33). Ultimately, nurse initiatives for leadership and effective communication during the pandemic will also determine their professional growth and status in the future (34).

### Situation in Pakistan

On-the-job training and continued learning for clinical nurses is overall a neglected topic in Pakistan (35). At the outbreak of the COVID-19 pandemic, the government of Pakistan initiated briefing sessions for infection control and awareness among healthcare practitioners. But not all the sessions have been comprehensive, ongoing or supportive for reinforcement, or with provision for questions and answers (36). Some local research has identified that only half of nurses received any formal training of infection prevention and control or how to manage COVID-19 cases (37). Further research highlighted that healthcare practitioners did not receive any support for learning or training. And even if they received some support, it only consisted of a briefing in a single session by a senior doctor or a placement of notes across wards by an infection control nurse (37). Furthermore, many practitioners have been provided written guidelines without instruction or specific guidelines.

In Pakistan, there is a critical need for nurses and other allied healthcare staff to receive continued learning support (38). Healthcare professionals have reported the need for clearer communication related to infection prevention and control (39). There is also a need for a more systematic and integrated infection control team at each hospital, with a trained infection control focal person responsible for ensuring prevention and standard practices meeting updated international guidelines (40). Training to nurses for leadership and communication also needs prioritization in Pakistan (41). Nurses have mostly been excluded as leaders in the clinical setting, with leadership training and leadership roles being allocated to physicians and hospital administration (42). In addition, most nurses report communication problems with their leaders and coworkers (43). Continued learning and training for nurses in leadership and communication must be initiated in order to secure high-quality healthcare services. The development of effective treatment plans during the pandemic and beyond is a necessity (44).

### Role of Social Media in Continued Learning

The constantly changing nature of providing and receiving information as well as uncertainty and inadequacy of training processes has made it imperative for nurses and other healthcare practitioners to have supplementary support for learning and knowledge building. A salient question during the COVID-19 pandemic is to ascertain how to support frontline practitioners like nurses for continued learning and pandemic preparedness (45). Improving training support requires a multi-pronged approach, with support coming in not just from hospital administration and the government, but also public health experts and the private sector. In fact, multiple platforms or methods of communication are preferred for receiving training and accessing information related to infection prevention and disease management (46). The long and erratic work hours during the coronavirus pandemic has necessitated that nurses be supported with different modes of training which suits their schedule (45). Single didactic training sessions with large number of participants have received less appreciation from participants. Prior research supports the effectiveness of employing a distributed practice approach for enhancing knowledge retention (47).

WhatsApp-based interventions to support learning can be beneficial as they allow participants to download and review material when they are free and relaxed (48). Some of the advantages of WhatsApp-based learning include (i) free choice of time during the day to absorb material and interact with questions, (ii) supporting a combination of training tools (text, audio, image, and video), and (iii) maintaining physical distancing during the pandemic. However, a recent health intervention has emphasized that WhatsApp moderators must be present to facilitate learning and communication between trainers and participants (49).

### Aims of Study

There is immense reliance on nurses with regard to patient safety. However, very little effort is made to invest in interventions for their continual learning support and leadership roles in developing countries, including Pakistan (12, 50). Empowering nurses through training during the COVID-19 pandemic is an essential step to facilitate nurse leadership and safety culture in hospitals (19, 51).

The overall scope of this research is to improve nurse leadership in maintaining minimum service delivery standards for hospital infection control with respect to COVID-19, but also other infectious disease burdens beyond the pandemic (52–54). We believe that research about potential benefits for on-going training for nurses and digital literacy options will develop broader options for training support for nurses in Pakistan and other developing regions. In lieu of this, the aim of the study is to deliver a digital intervention for continued learning to supplement and reinforce information for currently working nurses in the age of coronavirus with respect to three areas: (i) infection prevention and control, (ii) COVID-19-related knowledge, and (iii) leadership and communication. Improvement in learning and efficacy of the intervention will be assessed by making a comparative study of participant's knowledge base before and after the intervention and by comparison with a control group who has not received the intervention.

In this way, this study aims to investigate three research questions:

1. Is there a significant difference between the pre-post-test results of the intervention and control group after the intervention, in comparison to their baseline scores and in comparison to each other?
2. Do the socio-demographic characteristics of the nurse participants in the intervention group show significant associations with post-test results?
3. Is there any feedback about the value of the WhatsApp-based intervention from participants in the intervention group in order to plan future interventions?

## Methods

### Sample

We included nurses in our study who were employed in public or private hospitals of Lahore, Pakistan, during the COVID-19 pandemic. Nurses who were not employed at time of data collection were excluded from study participation. There were 81,816 nurses registered nurses in Punjab (55), with no confirmed or published data about the number of nurses in Lahore. We used the approximate population percentage of Lahore city, which is 10% of Punjab's population, to estimate a nurse population of 8,181 for Lahore. We then used the Taro Yamane formulae to estimate a sample size of 381 nurses. Lahore is the capital of the largest province of Pakistan, Punjab province, with an estimated population of above 11 million people. There are an approximate 15 private and 27 public hospitals in the city. All the sampled hospitals in this study belong to the Category 1 classification of teaching hospitals that have more than 500 beds and have been facilitating coronavirus patients in ICUs or separate wards. The focus in Category 1 hospitals secures the homogeneity of the sample and workplace environment. Four hospitals in total were sampled purposively, two each from the private and public sector. We were able to gain access to contact information (mobile number and email addresses) of between 111 and 203 nurses from each sampled hospital.

### Data Collection

We sent both phone and WhatsApp text messages to a total of 400 nurses, 100 randomly selected from each hospital, informing them about the research objectives and study design. A total of 372 nurses responded to the message and 344 agreed to participate in the study. We divided consenting participants by random alternate selection into the intervention group and control group with 172 participants each. By the end of the intervention, a total of 106 nurse participants remained part of the intervention group and 102 nurse participants from the control group returned the complete post-test survey.

Both the pre and post-test survey was administered through Google Survey Form, which is a free and safe method of collecting confidential data (56). Data were stored on a secure “cloud” database, where it was automatically sorted and scored. Each participant was allocated a code and no confidential data or names were shared. Data were held securely with the lead author (SRJ). The survey for this study was implemented on Google by one of the authors (SRJ) with a password protected account. The first set of questions on the survey included demographic data including the complete names of the nurses, their respective WhatsApp number and email address, name of lead intervention facilitator/WhatsApp moderator, WhatsApp group code, and hospital affiliation. This data was used to link the pre- and post-test data for each participant.

**Figure 1:**
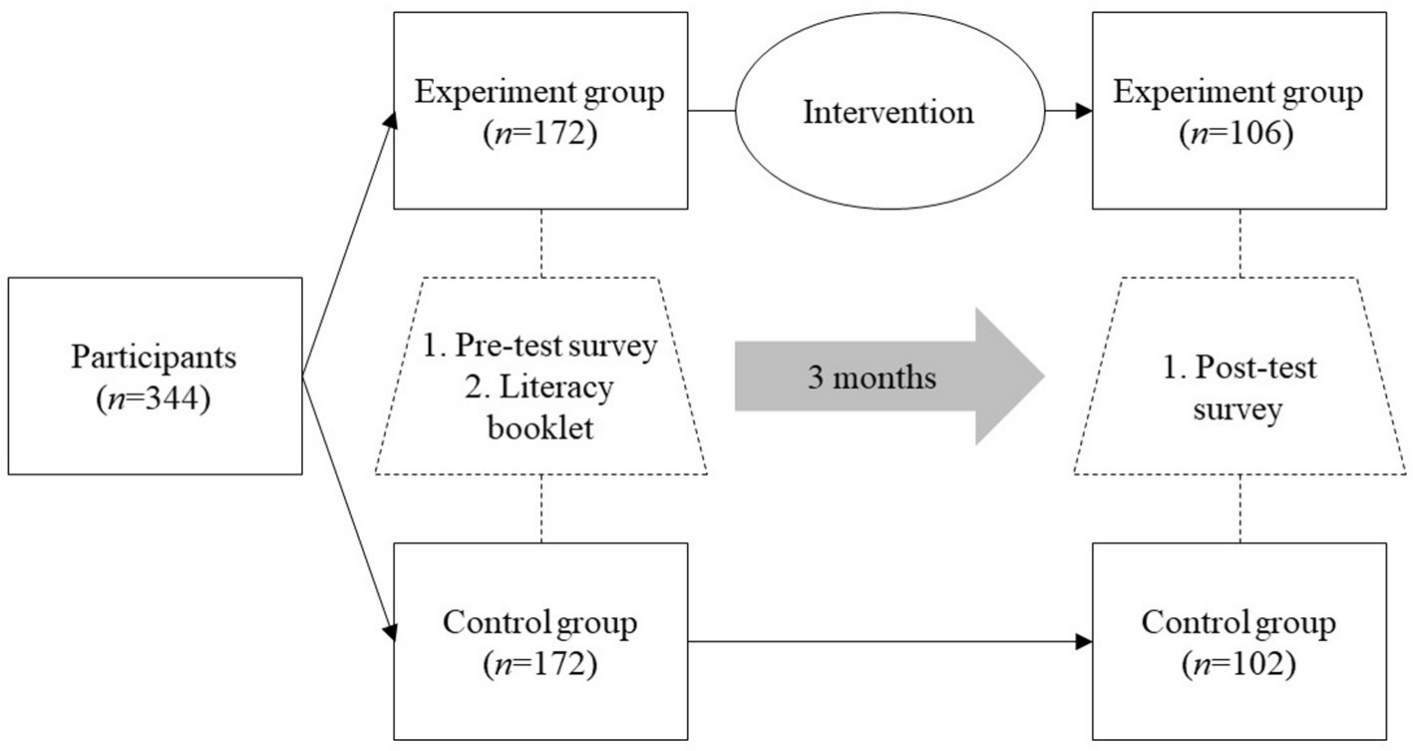
Flow of participants in the study.

The flow of nurse participants in the study is summarized in Figure 1. Reasons for participant drop-out were: (i) internet connectivity problems and cost of downloading videos on mobile data (*n* = 74; 54.4%), (ii) exit from group without response or explanation (*n* = 22; 16.2%), (iii) lack of time to be part of the intervention group and to read materials or watch videos (*n* = 15; 11.0%), (iv) self or family member infected with COVID-19 (*n* = 13; 9.6%), and (v) revoked permission from family members/husband to remain part of a WhatsApp group because of work-family conflict (*n* = 12; 8.8%).

### Intervention Design

The research design included administration of a pre-post-test survey and communication of a literacy booklet in English to both the control and intervention group. The intervention group received a more detailed intervention with learning information communicated *via* WhatsApp. The entire intervention lasted 12 weeks and included the following seven steps, which are also summarized in Table 1.

**Table 1:** Literacy modules instruction plan for intervention group.

**Steps**	**Days**	**Area**	**Content**
1	1–5	Pre-test survey	Supplementary Data Sheets 1–3
2	6–12	Continued learning booklet- Transferred in English and Urdu	- Three modules were part of the booklet: (i) infection prevention and control, (ii) COVID-19 knowledge, and (iii) leadership and communication.
		*Examples of questions to participants by trainers to encourage interaction:* - Did you manage to finish reading all three modules? - Did you find the material clear and useful? - Was the Urdu translation helpful in concept retention?	
3	13–25	Introductory Zoom session The principal investigators held one Zoom sessions, for 1 h each, with each of the 20 intervention groups as an introduction	- The study team, WhatsApp moderators, and trainers for continued learning were introduced. - There was discussion about learning objectives, aim of the intervention, and the importance of observing netiquettes. - The importance of participation in the exercises, case-study discussions, and feedback was stressed. - Each session covered a presentation summarizing the main areas of the three learning modules.
4	26–43	Video tutorials for infection prevention and control	- Introduction to infection prevention and control programmes - Structure and function of infection prevention and control programmes - Importance of on-going education of healthcare workers - Audits in infection prevention and control and audit tools - Policy development in infection prevention and control - Report writing in infection prevention and control - Occupational health and safety programmes
		*Examples of questions to participants by trainers to encourage interaction:* - What are the key indicators for infection prevention and control programmes? - What are the World Health Organisation's recommended core components for the implementation of infection prevention and control in health facilities?	
		*Case study discussion:* - Tuberculosis and HIV control programme - Airborne isolation precautions	
5	44–61	Video tutorials for COVID-19 knowledge	- Introduction to the virus, how it is spread, incubation period, and clinical diagnosis - How to prevent the spread - Mask safety and protective gear management - Clinical features of COVID-19 - Management of people with suspected COVID-19 infection - Management of patients with mild, moderate and severe COVID-19 - Protecting healthcare workers
		*Examples of questions to participants by trainers to encourage interaction:* - Can people who have recovered from COVID-19 become infected again? - What cleaning of surfaces and equipment is required in COVID-19 areas?	
		*Case study discussion:*- Managing an elderly COVID-19 patient with chronic illness - Advising asymptomatic people about self-isolation, self-monitoring and incubation period	
6	62–79	Video tutorials for leadership and communication	- Defining leadership at workplace - Characteristics and types of leaders - Components and channels of communication - Types of conflict - Managing conflict effectively to retain leadership
		*Examples of questions to participants by trainers to encourage interaction:* - Identify how characteristics and types of leadership relate to your own leadership approaches - Select the most effective channels of communication you have used at the workplace - Discuss which leadership skills and behaviors are needed for optimal conflict resolution.	
		*Case study discussion:*- Thomas-Kilmann Conflict Mode Instrument - Key characteristics preferred in focal leader at workplace	
7	80–84	Post-test survey	Supplementary Data Sheets 1–3

#### Step 1: Pre-test Survey

The pre-test survey was administered to both the control and intervention group through Google survey form. Each participant was sent a reminder through WhatsApp to complete the survey.

#### Step 2: Continued Learning Booklet

The booklet was sent through both email and WhatsApp individually to each participant in the control and intervention group. The continued learning booklet was distributed in English and Urdu language. It was developed based on a combination of relevant international standard guidelines. The booklet contained three modules with information related to the three study areas (Supplementary Data Sheets 1–3): (i) infection prevention and control (57, 58), (ii) COVID-19 knowledge (59, 60), and (iii) leadership and communication (61–63).

#### Step 3: Preliminary Zoom Sessions for Introduction (Intervention Group)

The principal investigators (SRJ, AJ, RRD, and SKB) of the study held an introductory Zoom session with the participants in the intervention group. The participants in the intervention group were divided in groups of 16–20 for the Zoom sessions, and the same groups were used to form the WhatsApp groups in the next step of the intervention. We encountered problems in coordinating a suitable time for group members. For that reason, for each group an attempt was made to hold at least three Zoom sessions to accommodate all participants and their requests for different time slots. During the Zoom introductory session with each group the following topics were discussed: (i) the objectives of the intervention in more detail, (ii) review knowledge of netiquette and observance of ethics in dealing with fellow participants during the intervention, (iii) elements of the content of the three modules, and (iv) introducing intervention deliverers/specialists in the field who would be sending video tutorials over the 12-week intervention period. The Zoom sessions were aimed to be a team building exercise to support participants to read the material and engage in feedback and Q&A sessions and also build a rapport with the WhatsApp facilitators. This Zoom session was also recorded and sent to each WhatsApp group.

#### Steps 4–6: WhatsApp Group Learning (Intervention Group)

**Figure 2:**
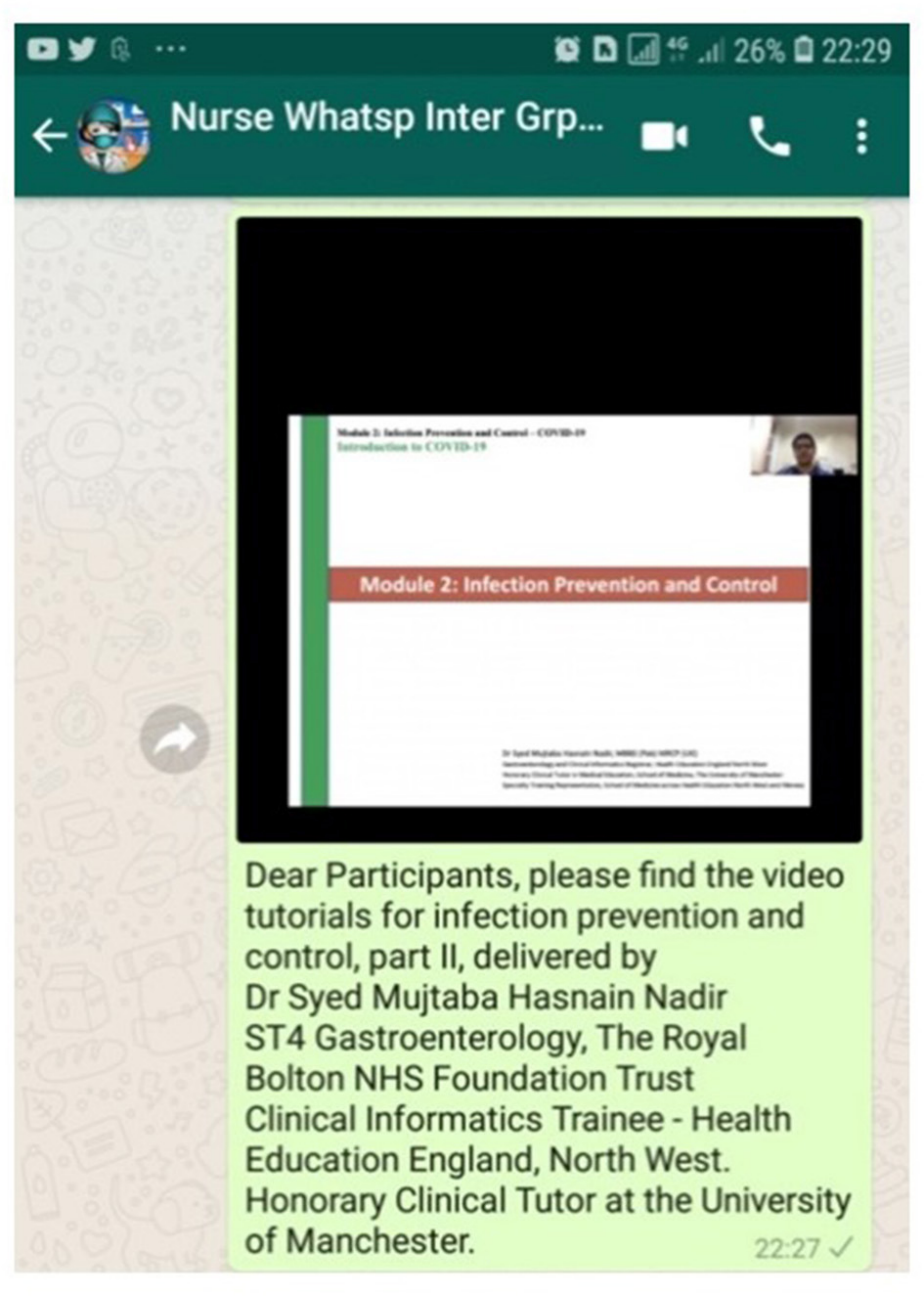
Screenshot of a WhatsApp communication to the intervention group including a video tutorial by subject expert and introductory text.

As mentioned above, 20 WhatsApp groups with 16–20 participants each were formed for the intervention group for the duration of the intervention. Each group was managed by one WhatsApp moderator and a team of health experts (three authors of this study-SH, FA, and AS), who were responsible for administration and support of participants through different hours of the day (Figure 2). The WhatsApp group was used to send text messages, PDF files, images, audio notes, and video tutorials related to the three learning areas of (i) infection prevention and control, (ii) COVID-19 knowledge, and (iii) leadership and communication. A total of 21 tutorials by five experts and clinicians, with 5–10 min duration each, were sent to the intervention group participants every 2 days. Including case-study discussion and time for question and answers, each of the three learning areas was covered over a period of 18 days each.

Daily messages were sent to provide support in answering questions of participants and coordinating feedback and comments about the training. Though nursing school education is conducted in English in Pakistan, Urdu is the national language of the country and the medium of instruction at primary and secondary level includes Urdu, English and the provincial language (64). It was thus deemed valuable to provide reinforcement by translating the literacy booklet material in Urdu to help in building memory blocks and retention of information.

#### Step 7: Post-test Survey

The post-test survey was administered to both the control and intervention group through Google survey form. Again, participants were individually sent a reminder through WhatsApp to complete the survey.

### Facilitators of the Intervention

A team of 20 nurses was recruited to support the project for (i) support to and coordination of the nurse participants for the study, (ii) creation and moderation of the WhatsApp groups, and (iii) referral of questions and answers between nurse participants and trainers for continued learning. All WhatsApp nurse moderators were registered and licensed nurses from Pakistan Nursing Council with at least 1 year of experience in the clinical setting. Two authors of this study (NR and SRJ) were responsible for a one-week training of the WhatsApp moderators for the objective of the study and how to facilitate the intervention.

The continued learning written material and video tutorials have been developed by six experts (RZ, SH, AM, FA, AS, and SKB) who had one or more of the following qualifications: (i) medical doctor currently working in the COVID-19 ward or isolation centers; (ii) trainer for infection prevention and control and coronavirus management; and/or (iii) trainer for public health safety and leadership and communication at the workplace.

### Pre- and Post-test-survey

The pre-post-test survey has been developed using items from the intervention material. A total of 42 questions were part of the survey, with 10 questions for socio-demographic characteristics, nine questions related to the workplace, and eight questions each for the three study intervention areas (“infection prevention and control,” “COVID-19 knowledge,” and “leadership and communication”). A five-point Likert scale was used with responses from “strongly disagree,” “disagree,” “neutral,” “agree,” and “strongly agree.” Coding ranged from “strongly disagree” being allocated a score of 1 to “strongly agree” being allocated a score of 5. Cronbach's alpha was calculated in reliability analyses for the three domain scales. All three scales showed good reliability (infection prevention and control: α = 0.770; COVID-19 knowledge: α = 0.746; leadership and communication: α = 0.759).

### Data Analysis

#### Quantitative: Statistical Analysis

The hypotheses for this study are:

H1: Nurses in the intervention group, compared to the control group, report an increase in knowledge in the three study intervention areas: (i) infection prevention and control, (ii) COVID-19 knowledge, and (iii) leadership and communication.

H2: Nurses in the intervention group report an increase in knowledge compared to their baseline scores, in the three study intervention areas: (i) infection prevention and control, (ii) COVID-19 knowledge, and (iii) leadership and communication.

H3: Socio-demographic characteristics of the intervention group are associated with improved learning areas in the post-intervention scores.

Data analysis was performed to determine the main effect of the intervention using SPSS 25.0. Descriptive statistics were used to describe the characteristics of the control and intervention group. One-way ANOVA was performed to assess the relationship between socio-demographic characteristics with each of the three study intervention areas (“infection prevention and control,” “COVID-19 knowledge,” and “leadership and communication”) at baseline. Paired sample *t*-tests were run to compare the post intervention mean differences between the control and intervention group for each item used to measure the three study domains.

The three study domains were compounded for further analysis with five points on each item; with the highest score for satisfactory learning and knowledge. Respective compounded scores for each of the three domains are considered as dependent variables for the analysis. Independent sample *t*-tests were used to compare the mean difference between the control and intervention group for the three study intervention areas. Finally, ANCOVA was performed to assess mean differences post intervention holding confounding variables (baseline results, age, gender, income, years of work, degree type, and type of contract) constant. All *p* < 0.05 were considered significant for this study.

#### Qualitative: Content Analysis

The WhatsApp conversations were transferred to Microsoft Word for coding of themes. A total of 1,368 messages were received from nurse participants through the 12-week intervention. Independent manual coding (65) was performed by two of the authors (RD and SJ). At the first step, all emoticons were excluded (examples of these emoticons include thumbs up, clapping hands, raising hands, folded hands, and smiling face). Next, common thematic categories were grouped together based on frequency. There were some messages typed in Urdu, which were translated into English. Translations were doubled-checked by a third author (SKB) through the forward backward method (66). Authors held meetings to confirm and corroborate final themes. Final themes were also discussed with two the WhatsApp moderators and 10 nurse participants for further verification (67).

### Ethics

Ethics approval for this study was taken from the Institutional Review Board, Forman Christian College (A Chartered University). All ethics were observed diligently with respect to informed consent and confidentiality of nurse participants. No names of nurses and hospitals have been reported. Participants were not provided any incentive to participate in the study and they were informed about the freedom to exit the intervention if and when they chose.

We ensured that all participants who were grouped together in one WhatsApp group (not more than 20 participants) were from the same hospital and had a common employer. In this way, we were able to guarantee both consent of participants and safety with regard to data security and privacy protection (68).

## Results

### Socio-Demographic Characteristics

The socio-demographic characteristics of the sample are presented in Table 2. The majority of respondents are aged below 39 years and female. ANOVA results show homogeneity of the control and intervention group at baseline (Supplementary Material 1).

**Table 2:** Descriptive statistics of the sample (*n* = 208).

	**Control group (*n =* 102)%**	**Intervention group (*n =* 106)%**	*p*-value^*^
Age (in years)			
20–29	86.3	69.8	**0.007**
30–39	13.7	26.4	
40+	–	3.8	
Gender			
Female	65.7	80.2	**<0.001**
Male	34.3	19.8	
Type of contract			
Permanent	70.6	60.4	0.122
Temporary	29.4	39.6	
Years of service			
0–5	63.7	42.5	**<0.001**
≥ 6	36.3	57.5	
Monthly income			
< PKR 59,000/USD 383.7	82.4	55.7	**<0.001**
≥PKR 59,000/USD 383.7	17.6	44.3	
Last degree			
Diploma	62.7	51.9	**0.034**
BSc or above	37.3	48.1	
Marital status			
Single	73.5	61.3	0.061
Currently married	26.5	38.7	

* *p-value was calculated by Chi square statistics; p-values are considered significant at <0.05. Bold values indicate significant results*.

### Pre- and Post-intervention Mean Results for the Intervention Areas

Table 3 presents the pre- and post-intervention mean results for the intervention and control group with respect to the three study intervention areas. At post-intervention, the mean values for the intervention group, compared to the control group, are higher for all intervention areas: (i) “infection prevention and control” (Mean difference: 1.11); (ii) “COVID-19 knowledge” (Mean difference: 1.84); and (iii) “leadership and communication” (Mean difference: 1.45). Additionally, all three study domains show significant results after the intervention.

**Table 3:** Mean results for the three intervention areas for the control and intervention group at pre- and post-intervention (*n* = 208).

	**Pre-intervention (M ±SD)**	***T***	*p*-value^*^	**Post-intervention (M ±SD)**	***t***	*p*-value^*^
Infection prevention and control						
Intervention group	31.77 ± 0.55	2.709	**0.007**	34.36 ± 2.68	2.748	**0.007**
Control group	31.47 ± 1.00			33.25 ± 3.14		
COVID-19 knowledge						
Intervention group	31.6 ± 0.79	1.733	0.085	34.97 ± 6.13	2.641	**0.009**
Control group	31.44 ± 1.29			33.13 ± 3.47		
Leadership and communication						
Intervention group	31.60 ± 0.95	0.528	0.598	33.54 ± 2.98	3.435	**0.001**
Control group	31.52 ± 1.07			32.09 ± 3.10		

* * p-values are considered significant at <0.05. Bold values indicate significant results*.

### Post-intervention Results for Items of the Intervention Areas

Table 4 presents the post-intervention results for each item [means (M) and standard deviations (SD)] of the three intervention areas (“infection prevention and control,” “COVID-19 knowledge,” and “leadership and communication”). For “infection prevention and control,” the following items show greater mean values and significant results in the intervention group: (i) Knowledge of what is part of infection prevention and control (*p* = 0.001); (ii) Confidence in knowledge about controlling infection at the workplace (*p* = 0.040); (iii) Confidence in collaboration with other healthcare workers for infection prevention and control (*p* = 0.044); (iv) Confidence in contributing to written records for infection prevention and control (*p* = 0.019).

**Table 4:** Mean results for individual items measuring the intervention area for the control and intervention group at post-intervention (*n* = 208).

	**Control group (M ±SD)**	**Intervention group (M ±SD)**	**Mean difference**	***t***	*p*-value^*^
Infection prevention and control					
Knowledge of what is part of infection prevention and control	4.29 ± 0.55	4.55 ± 0.55	0.26	3.41	0.001
Confidence in knowledge about controlling infection at the workplace	4.20 ± 0.63	4.37 ± 0.55	0.17	2.06	0.040
Confident in collaboration with other healthcare workers for infection prevention and control	4.15 ± 0.50	4.31 ± 0.57	0.16	2.02	0.044
Knowledge regarding audit for infection prevention and control	4.22 ± 0.71	4.25 ± 0.63	0.03	0.31	0.755
Knowledge of occupational health and safety generally	4.06 ± 0.58	4.14 ± 0.62	0.08	0.86	0.386
Confidence in contributing to written records for infection prevention and control	4.11 ± 0.63	4.31 ± 0.54	0.2	2.36	0.019
Confidence in supporting improvement in structure and function of infection prevention and control at hospital	4.09 ± 0.59	4.21 ± 0.53	0.12	1.50	0.133
Thinking about contributing to policy development for infection prevention and control	4.08 ± 0.64	4.19 ± 0.59	0.11	1.27	0.202
COVID-19 knowledge					
Knowledge regarding COVID-19 infection and spread	4.21 ± 0.75	4.45 ± 0.64	0.24	2.43	0.016
Knowledge regarding COVD-19 diagnosis and vaccination development	4.15 ± 0.57	4.33 ± 0.61	0.18	1.99	0.048
Knowledge regarding COVID-19 prevention and protection	4.18 ± 0.68	4.87 ± 0.94	0.69	1.39	0.163
Ability to advise others about COVID-19 prevention and protection	4.08 ± 0.73	4.32 ± 0.56	0.24	2.57	0.011
Ability to advise my employer and government about COVID-19	4.23 ± 0.52	4.32 ± 0.52	0.09	1.16	0.245
Ability to manage people with suspected COVID-19 infection	3.97 ± 0.81	4.20 ± 0.65	0.23	2.31	0.022
Ability to manage people with mild COVID-19 infection	4.14 ± 0.58	4.30 ± 0.55	0.16	1.95	0.050
Ability to manage people with moderate or severe COVID-19 infection	4.13 ± 0.64	4.16 ± 0.71	0.03	0.34	0.730
Leadership and communication					
Knowledge in defining skilled nurse leadership	3.91 ± 0.75	4.24 ± 0.56	0.33	3.60	<0.001
Ability to assume leadership	4.00 ± 0.62	4.18 ± 0.58	0.18	2.23	0.026
Knowledge of the key roles and tasks that a focal focal nurse professional is responsible for	4.08 ± 0.66	4.12 ± 0.69	0.04	0.36	0.716
Confident in ability to assume a position as a focal nurse professional	4.02 ± 0.47	4.17 ± 0.62	0.15	1.93	0.055
Knowledge of the different components and channels for communication at the workplace	4.04 ± 0.60	4.26 ± 0.60	0.22	2.56	0.011
Confidence in ability to communicate more effectively at work	3.94 ± 0.65	4.12 ± 0.61	0.18	2.06	0.041
Knowledge regarding conflict management at workplace	4.07 ± 0.74	4.36 ± 0.55	0.29	3.19	0.001
Confident in my ability to manage conflict at work	4.00 ± 0.67	4.05 ± 0.59	0.05	0.64	0.523

* *p-values are considered significant at <0.05*.

For “COVID-19 knowledge,” the following items show greater mean values in the intervention group with significant results: (i) Knowledge regarding COVID-19 infection and spread (*p* = 0.016); (ii) Knowledge regarding COVID-19 diagnosis and vaccination development (*p* = 0.048); (iii) Ability to advise others about COVID-19 prevention and protection (*p* = 0.011); (iv) Ability to manage people with suspected COVID-19 infection (*p* = 0.022); and (v) Ability to manage people with mild COVID-19 infection (*p* = 0.050).

For “leadership and communication,” the following items show greater mean values and significant results in the intervention group: (i) Knowledge in defining skilled nurse leadership; (*p* < 0.001); (ii) Knowledge regarding conflict management at workplace (*p* = 0.001); (iii) Knowledge of the different components and channels for communication at the workplace (*p* = 0.011); (iv) Confidence in ability to communicate more effectively at work (*p* = 0.041); and (v) Ability to assume leadership (*p* = 0.026).

### Stratification of Post-intervention Results for the Intervention Group

Table 5 presents the mean results at post-intervention for the intervention group stratified by socio-demographic characteristics. There are no significant results associated with socio-demographic characteristics of participants in the intervention group, except for one area: Under the intervention area of “leadership and communication,” we find a significantly higher mean in improved learning for nurses with BSc Degrees compared to nurses who are Diploma holders (Mean difference:1.28).

**Table 5:** Comparative means by socio-demographic characteristics of the intervention group at post-intervention (*n* = 106).

	**Infection prevention and control**			**COVID-19 knowledge**			**Leadership and communication**		
	**M ±SD**	***F* stat (df)**	*p*-value^*^	**M ±SD**	***F* stat (df)**	*p*-value^*^	**M ±SD**	***F* stat (df)**	*p*-value^*^
Age									
20–29 years	34.50 ± 2.72	0.50 (2, 103)	0.608	34.27 ± 7.09	0.29 (2, 103)	0.748	33.71 ± 3.08	0.39 (2, 103)	0.674
30–39 years	34.17 ± 2.70			34.32 ± 3.03			33.71 ± 2.66		
40+ years	33.25 ± 1.89			34.00 ± 1.63			33.00 ± 3.74		
Gender									
Female	34.34 ± 2.60	0.42 (2, 104)	0.838	35.24 ± 6.70	0.86 (1, 104)	0.355	33.75 ± 3.07	2.06 (1, 104)	0.154
Male	34.47 ± 3.07			33.85 ± 2.66			32.71 ± 2.47		
Type of contract									
Permanent	34.06 ± 2.53	2.17 (1, 104)	0.150	34.53 ± 3.02	0.83 (1, 104)	0.364	33.71 ± 2.87	0.53 (1, 104)	0.467
Temporary	34.83 ± 2.87			35.64 ± 9.02			33.28 ± 3.16		
Years of service									
0–5 years	34.00 ± 2.68	0.01 (1, 104)	0.891	33.00 ± 6.13	0.10 (1, 104)	0.748	35.00 ± 2.98	0.23 (1, 104)	0.627
>6 years	34.37 ± 2.70			34.99 ± 6.15			33.53 ± 2.99		
Monthly income									
< USD 383.7	34.45 ± 2.69	0.14 (1, 104)	0.702	35.05 ± 7.74	0.02 (1, 104)	0.882	33.52 ± 2.93	0.07 (1, 104)	0.933
≥USD 383.7	34.25 ± 2.70			34.87 ± 3.18			33.57 ± 3.06		
Last degree									
BSc or above	34.63 ± 2.63	1.14 (1, 104)	0.18	34.70 ± 8.10	1.66 (1, 104)	0.200	34.16 ± 3.22	5.07 (1, 104)	0.026
Diploma	34.07 ± 2.74			34.17 ± 2.59			32.88 ± 2.56		
Marital status									
Single	34.38 ± 2.55	0.06 (1, 104)	0.936	35.33 ± 7.43	0.59 (1, 104)	0.441	33.53 ± 2.95	0.01 (1, 104)	0.970
Currently married	34.34 ± 2.92			34.39 ± 3.11			33.56 ± 3.05		

* *p-values are considered significant at <0.05*.

### ANCOVA

The ANCOVA results for the intervention and control group at post-intervention stage holding confounding variables (baseline results, age, gender, income, years of work, degree type, and type of contract) constant, is presented in Table 6. A statistically significant result reveals a higher mean score for the intervention group (34.36 ± 2.68) compared to the control group (33.25 ± 3.14) for the intervention area of “infection prevention and control.” The intervention area of “leadership and communication” also shows a higher mean score for the intervention group (33.54 ± 2.98) compared to the control group (32.09 ± 3.10). Though the mean results are higher for the intervention group compared to the control group for learning in “COVID-19 knowledge,” the results are not significant.

**Table 6:** ANCOVA results for the three study domains for the control and intervention group after controlling for confounding variables (*n* = 208).

	**Post-intervention (M ±SD)**	**Adjusted mean difference (95% CI)**	*F* stat (df)^*^	*p*-value^**^
Infection prevention and control				
Intervention group	34.36 ± 2.68	1.17 (−0.17 to 2.11)	5.38 (7, 198)	0.021
Control group	33.25 ± 3.14			
COVID-19 knowledge				
Intervention group	34.97 ± 6.13	0.02 (−1.63 to 1.69)	3.50 (7, 200)	0.063
Control group	33.13 ± 3.47			
Leadership and conflict management				
Intervention group	33.54 ± 2.98	1.00 (−2.01 to 0.00)	3.85 (7, 200)	0.050
Control group	32.09 ± 3.10			

* *ANCOVA confounding variables held constant including baseline results, age, gender, income, years of work, degree type, and type of contract*.

** *p-values are considered significant at <0.05*.

### Content Analysis

Intervention participant responses counted from the WhatsApp group included a total of 1,368. Four main thematic responses of the intervention group were identified: (i) Appreciation for the intervention (772 messages, 56%); (ii) Specific questions for instructors (284 messages, 21%); (iii) Request for more information or WhatsApp learning interventions (232 messages, 17%); and (iv) Feedback to improve the intervention (80 messages, 6%). Table 7 summarizes these responses and also includes direct quotes of participants.

**Table 7:** Themes from the content analysis describing intervention group participant interaction.

**Theme (frequency)**	**Examples of quotes**
Appreciation (772 messages)	- “Very good effort to define coronavirus and its prevention” - “These are informative videos which are helping us in our routine work” - “I am reading the material again and find it useful during this second wave of COVID-19” - “Thanks for being available even at midnight to answer questions” - “The data and guidelines shared have been very useful…it has been a wonderful experience. I will miss the group” - “Good content has been shared, especially the material related to conflict management” - “Videos and modules are very informative and helpful and have surely increased my knowledge for infection control and coronavirus” - “Useful information regarding infection control measures has been shared which can help us save lives”
Specific questions for instructors (284 messages)	- “How do we motivate illiterate populations and family attendants to mask up?” - “In case of RTA (road traffic accidents) where we need to do CPR and give breaths…and we don't know if patient is COVID-19 positive should we proceed with CPR…what are the other options?” - “If someone is infected and asymptomatic how do we prevent the spread?” - “Are asymptomatic patients less contagious and are presymptomatic people less contagious?” - “Which population is at more risk in this pandemic?” - “How do we deal with compliance in illiterate populations”? - “What is your opinion about how long coronavirus will continue” - “Why is coronavirus not affecting children as much as adults…will this change with development of new variants?” - “Can pregnant and lactating mothers get the vaccine?” - “Can we get COVID-19 even after getting both doses of the vaccine?” - “Can we get the coronavirus vaccine if we have cold or fever or should we wait?”
Request for more information or interventions (232 messages)	- “Please have another group for clinical information” - “I like that we can read and watch the videos at home at night when we are relaxed and the children are asleep” - “I prefer that in this WhatsApp group we are able to move back and forth in Urdu. This is better than the conferences which are conducted in English, with little time for Q&A sessions.” - “WhatsApp learning is better. What I like is that we can think about the material and video content for a few days and read/ watch them again. We then have time to ask questions later.” - “Please have another intervention like this for emotional learning and conflict management” - “Please include administrators in trainings as they influence work environment…which in turn influences our practices.”
Feedback (80 messages)	- “There was no need for the Urdu module” - “The booklet in English was enough. Because we have studied these things in English” - “The videos and content related to conflict management must be passed on to our supervisors. It would be better to have sessions with them.” - “Reading about conflict management is useless, unless our leaders are trained first, including doctors and hospital administration.” - “Infection control has a lot to do with the working environment and administrative support for resources. Training the public and family attendants is also needed.” - “We cannot manage infection alone. We need support from our administration for resources and PPE Kits.”

## Discussion

To the best of our knowledge, this is the first study to attempt delivering a digital literacy intervention for continued learning through WhatsApp to practicing nurses during the COVID-19 pandemic in Pakistan. The results suggest that online digital learning, and WhatsApp specifically, provides convenient and effective support for continued learning in nurses as an additional tool to advance literacy, communication, and skill development.

Adherence to the International Council of Nurses International Code of Ethics implies that all nurses, regardless of designation, and demographic differences, are committed to maintaining competency through continual learning (69). With respect to the first hypothesis of our study, the results confirm that nurses in the intervention group, compared to the control group, do report an increase in knowledge in the three areas of intervention delivery (“infection prevention and control,” “COVID-19 knowledge,” and “leadership and communication”). Previous research confirms the effectiveness of online-based digital interventions for nurses and also argues for blended training combining digital and in-person support (70). Our findings also corroborate further research which suggests that digital interventions can strengthen the workforce for infection control practices and consequently improve health system performance at macro-level (71).

Findings also identify the specific areas where the intervention group, compared to the control group, report improved knowledge about COVID-19. Our results suggest that online-based digital literacy interventions for COVID-19 knowledge and management have an effect on the confidence and perceived ability of practitioners. Other research suggests that nurse literacy interventions about epidemiology of disease and patient management have an impact on improved clinical practices and participation in improved care planning for patients (72). Furthermore, recent research confirms that online-based digital interventions have the benefit of rapid deployment of educational material and awareness in frontline healthcare providers (73).

Results also ascertained specific areas of improvement in the intervention group, compared to the control group, under the intervention area of “leadership and communication.” Findings reveal that participants from the intervention group have improved knowledge in defining skilled nurse leadership and differentiating components and channels for communication at the workplace. The intervention group was also able to identify better practices for conflict management at the workplace and communicate more effectively at work. Overall, our results suggest that learning for leadership and communication through online-based digitals platforms are valuable and can be delivered in parallel to other online interventions being currently planned for the psychological wellbeing of nurses (74).

With regard to the second hypothesis of the study, univariate results confirm that the intervention group does report significant increases in knowledge compared to their baseline scores for the three study intervention areas. However, holding confounding variables constant, ANCOVA results show that the intervention group shows significantly improved results for only two study intervention areas, which are “infection prevention and control,” and “leadership and communication.” Results confirm that online WhatsApp interventions to improve nurse management are of value during the pandemic and in times of emergency. It is possible that the “COVID-19 knowledge” intervention area is not significant, because healthcare workers need additional, long-run, and continuous learning support for this area, especially in developing regions where government response and information sharing is weak (75). Even frontline practitioners from the developed world are suffering from low confidence in managing COVID-19 patients due to the uncertainty of the disease and evolving knowledge about variants (76).

Results for the third hypothesis of the study suggest that there is no significant association with socio-demographic characteristics of intervention participants and intervention areas. In this way, regardless of designation and social background, intervention participants show homogenous learning outcomes. The International Code of Ethics, outlined by the International Council of Nurses, also suggests that all nurses, regardless of designation and demographic differences, are committed to maintaining competency through continual learning (69). However, under the intervention area of “leadership and communication,” we found one significant demographic characteristic for improved learning in BSc Degree graduates compared to the Diploma holders. This finding implies that BSc degree holders absorb or value literacy and knowledge related to leadership and communication more. It may also be that BSc holders feel they have a better chance in practicing leadership and having opportunities for leadership positions in the future, thus the improved learning. Other scholars have argued that the Pakistan Nursing Council and Higher Education Commission must ensure additional training, capacity building, and minimum standards for the 3-year Nursing Diploma program (35). There is also concern that unless nurses are supporting in both training and career enhancement it can contribute to nurse stress and poor service delivery (77).

Finally, content analysis of the responses of the intervention participants during the intervention revealed that the majority appreciated the training and opportunity for continued learning through WhatsApp. Approximately one-fifth requested for similar interventions to be repeated and some requested specifically for more training under the area of communication and conflict management. A significant number also remained involved during the intervention in asking questions related to the virus, vaccination, and how to support vulnerable populations. The main feedback for change in the intervention was to ask for group trainings with other healthcare professionals, specifically supervisors. There was also request to include hospital administration in training for infection control in order to mobilize key reform in hospital environment and supply of resources.

### Limitations

There are some limitations to this study. The sample size was small and restricted to nurse participants from one city. However, findings may be generalizable for teaching hospitals of category 1 across the country as the health regulatory departments at provincial level follow the same guidelines. Similarly, we hope that training and continued learning reforms in major cities like Lahore will be replicated in other cities. There was a high dropout during the intervention period, mainly due to lack of time and also internet speed issues. Mean results for “COVID-19 knowledge” may not be representative for long-term perceptions of literacy as the epidemiology of the disease evolves. There is scope to repeat this study with a bigger sample, longer intervention period, and to include other healthcare practitioners.

## Conclusions

WhatsApp education and learning interventions for frontline healthcare practitioners show promise due to the convenience of at-hand, self-paced, and interactive sessions that uses multiple mediums. Participants in the intervention group of our study reported significant improvement in learning after the intervention for “infection prevention and control,” “COVID-19 knowledge,” as well as “leadership and communication.” Findings add to existing research that nurses require longer periods of training and support for continued learning and not just training that is restricted to a few hours or 2–3 days. It is also important that even during pandemics and physical distancing, we must continue online-based digital learning for practicing frontline workers to support a learning mindset without breaks in skill development and knowledge building. Since nurses with BSc backgrounds show improved learning in “leadership and communication,” we recommend skill development and certificate equivalency for Diploma holding nurses in Pakistan to support their learning ability and overall professional development and care delivery practices.

Our results provide impetus for health educators and practitioners who are planning training and interventions to use WhatsApp and other digital literacy interventions in times of pandemics and other health crises for ongoing and immediate communication. We recommend stakeholders like the health ministry, employers and hospital administration to conduct or outsource training through digital platforms as a more cost effective and time-saving means of continued learning. We also recommend more interdisciplinary and interprofessional online-based training between administration, allied health staff, and senior healthcare practitioners to support continued learning, enhanced communication, and improved patient safety practices. This study also makes a case for bilingual training for nurses. It is important for nurses to receive training and be comfortable in delivering care and talking to their patients in their local language with respect to treatment plan and infection management. Finally, we recommend that WhatsApp health education trainings can become more popular if (i) there are improved options for privacy protection of participants, with group administrators being able to conceal contact information of participants, and (ii) on the completion employers and health institutes can provide collaborative support for participant certificates that are recognized.

## Data Availability Statement

The raw data supporting the conclusions of this article will be made available by the authors, without undue reservation.

## Ethics Statement

The study was reviewed and approved by the Institutional Review Board, Forman Christian College. The participants provided their written informed consent to participate in this study.

## Author Contributions

SJ: conceptualization, methodology, and writing—original draft preparation. SJ, AJ, RD, and QM: formal analysis. SJ, NR, SB, RZ, SH, AM, FA, and AS: investigation. RZ, KJW, QM, and FF: writing—review and editing. RZ and FF: supervision. All authors have read and agreed to the published version of the manuscript.

## Funding

This project has been funded by Shahid Hussain Foundation. The grant (PKR 400,000/ USD 2,672.26) has been used to pay the nurse intervention facilitators for assistance in sampling and moderation of WhatsApp groups. Twenty nurse WhatsApp moderators/intervention facilitators have been paid PKR 20,000/ USD 131.36 each for this project directly through Shalamar Hospital Finance Office. Principal Investigators of the project did not receive any remuneration for this project. We acknowledge support for the publication costs by the Open Access Publication Fund of Bielefeld University.

## Conflict of Interest

The authors declare that the research was conducted in the absence of any commercial or financial relationships that could be construed as a potential conflict of interest.

## Publisher's Note

All claims expressed in this article are solely those of the authors and do not necessarily represent those of their affiliated organizations, or those of the publisher, the editors and the reviewers. Any product that may be evaluated in this article, or claim that may be made by its manufacturer, is not guaranteed or endorsed by the publisher.
