# Supplementary material for: WhatsApp-Delivered Intervention for Continued Learning for Nurses in Pakistan During the COVID-19 Pandemic: Results of a Randomized-Controlled Trial
Source: Front Public Health. 2022 Feb 15;10:739761. doi: 10.3389/fpubh.2022.739761 (PMC8885589; doi:10.3389/fpubh.2022.739761)
Supplement: Supplementary file 1 [file Data_Sheet_1.pdf]

Intervention Training Material for Nurse Leadership Study

Module 1: Role and Structure of Infection Prevention and Control

Contents

Intervention Training Material for Nurse Leadership Study 1
Module 1: Role and Structure of Infection Prevention and Control 1
Objectives 4
Introduction to infection prevention and control programmes 5
1-1 What is infection prevention and control? 5
1-2 What is an infection prevention and control programme? 5
1-3 What is included in infection prevention and control programmes? 5
1-4 Why are infection prevention and control programmes needed? 6
1-5 Why are infection prevention and control programmes especially important in low-resource settings? 6
1-6 What are the main activities of an infection prevention and control practitioner? 6
1-7 What are the key indicators for infection prevention and control programmes? 7
Structure and function of infection prevention and control programmes 8
1-8 What is the structure of infection prevention and control programmes at different levels? 8
1-9 Who should take responsibility for the infection prevention and control programme? 8
1-10 What are the minimum requirements for infection prevention and control programmes? 8
1-11 What are the World Health Organisation's recommended core components for the implementation of infection prevention and control in health facilities? 8
1-12 How should healthcare facilities implement improvements to their infection prevention and control programme? 10
1-13 How can healthcare facilities assess and measure improvements in an infection prevention and control programme? 10
1-14 What is an infection prevention and control team? 10
1-15 How many infection prevention and control nurse practitioners are needed? 11
1-16 What is an infection prevention and control committee? 11
1-17 What legislation supports infection prevention and control programmes? 12

Education of healthcare workers 13
1-18 Why should healthcare workers be educated about infection prevention and control? 13
1-19 Which categories of healthcare workers should be trained in infection prevention and control? 13
1-20 When and how often should healthcare workers be trained in infection prevention and control? 13
1-21 Which education methods can be used to train staff in infection prevention and control? 14
1-22 Who should provide training of healthcare workers in infection prevention and control? 14
Audits in infection prevention and control 15
1-23 What is an infection prevention and control audit? 15
1-24 What is the purpose of infection prevention and control audits? 15
1-25 Who should conduct infection prevention and control audits? 15
1-26 What is needed to conduct an audit on infection prevention and control practice? 15
1-27 How and to whom should the audit findings be reported? 16
1-28 What infection prevention and control audit tools are available? 18
Policy development in infection prevention and control 19
1-29 What is a policy? 19
1-30 When is a policy needed? 19
1-31 How are policies helpful in improving infection prevention and control? 19
1-32 Who should be involved in drafting IPC policies? 19
1-33 What is involved in the process of drafting IPC policies? 20
1-34 Which components should be included in a policy? 20
1-35 How should new IPC policies be communicated and implemented? 21
1-36 What is a standard operating procedure (SOP)? 21
1-37 When is a standard operating procedure needed? 22
1-38 What is a guideline? 22
Report writing in infection prevention and control 23
1-39 What is an infection prevention and control report? 23
1-40 What should be included in the report? 23
Occupational health and safety programmes 26
1-41 What is an occupational health and safety (OHS) programme? 26

1-42 What is the link between infection prevention and control (IPC) and occupational health and safety (OHS) programmes? 26

1-43 Which diseases and activities of the occupational health and safety programme have relevance to infection prevention and control? 26

1-44 How should needle-stick injuries be handled? 26

1-45 How can needlestick injuries be prevented? 27

Case Studies 29

Case study 1 29

Case study 2 30

Case study 3 31

Case study 4 33

Adapted from: 34

1. Book - Infection Prevention and Control Paperback – August 23, 2020 edition by Dr Angela Dramowski (Author), Prof David Woods (Author), Prof Shaheen Mehtar (Author) . 34

Objectives

When you have completed this chapter you should:

1. Understand the role and structure of infection prevention and control (IPC) programmes
2. Be able to describe key indicators of IPC programmes
3. Know the basics of IPC audits
4. Know how to draft IPC policies and reports
5. Understand the relationship between IPC and occupational health and safety programmes.

Introduction to infection prevention and control programmes

1-1 What is infection prevention and control?

Infection prevention and control (IPC) aims to prevent or control the spread of infections in healthcare facilities and the community. IPC is a universal discipline with relevance to all aspects of healthcare. It is part of every healthcare workers' duty of care to ensure that no harm is done to patients, visitors or staff. All healthcare workers require at least a basic understanding of IPC principles and practice.

1-2 What is an infection prevention and control programme?

IPC programmes include activities, procedures and policies designed to reduce the spread of infections, usually within healthcare facilities. The primary goals of an IPC programme are:

- To prevent susceptible patients acquiring pathogenic (disease-causing) micro-organisms
- To limit the spread of antimicrobial resistant infections.

1-3 What is included in infection prevention and control programmes?

There are several components that are common to IPC programmes worldwide including:

- Skilled IPC practitioners (usually nurses, occasionally doctors) who co-ordinate the IPC programme activities and develop, revise, audit and implement policies
- Accountability for IPC and integration of IPC as an essential part of healthcare with direct links to clinical services and non-clinical services (e.g. healthcare facility management and support services).
- A mandate to implement best-practice standards and guidelines
- A strong education component, involving all categories of healthcare workers
- Surveillance for healthcare-associated infections and outbreaks.

Although the basic principles of IPC apply globally, each country and individual healthcare facility will need to adapt and add to the core elements based on their specific circumstances, e.g. differences in patient population, infectious disease profiles, and type of healthcare services delivered.

1-4 Why are infection prevention and control programmes needed?

Healthcare facilities are places where sick people congregate, creating many opportunities for micro-organisms to spread between patients, visitors and healthcare workers. Medical care is also increasingly complex, with multiple, invasive procedures increasing the risk of developing healthcare-associated infections (HAI). Many of these infections (up to 70%) are preventable. Research has proven that IPC programmes can make healthcare safer and more affordable by preventing the suffering, loss of life and cost caused by healthcare-associated infection.

1-5 Why are infection prevention and control programmes especially important in low-resource settings?

Many low-resource settings have a high burden of infectious diseases, including HIV, Hep B, Hep C and tuberculosis. IPC has a critical role to play in these settings to enhance patient safety and to avoid the use of scarce resources for the treatment of healthcare-associated infection.

1-6 What are the main activities of an infection prevention and control practitioner?

The main activities performed by the IPC practitioner include:

- Organising surveillance for healthcare-associated infections
- Providing advice and leadership in outbreak investigation
- Developing and delivering training on IPC to healthcare workers
- Developing and implementing IPC-related policies and procedures

- Auditing the quality and effectiveness of healthcare facility environmental cleaning
- Auditing the quality and effectiveness of disinfection and sterilisation practices
- Implementing local, national or international best-practice guidelines for prevention of infection transmission in clinical care.

In many countries, the IPC practitioner has other duties such as seeing to occupational health or quality management. The term ‘quality management’ refers to all activities related to quality planning, assurance, quality control and improvement. In some cases, this may hamper their ability to perform all the required IPC activities. Since the aim is to prevent harm to patients and staff, IPC programmes often form part of a healthcare facility’s quality management programme.

1-7 What are the key indicators for infection prevention and control programmes?

There are three key indicators that can be used to report on the impact of an IPC programme.

1. Compliance indicators: these rate how well local or national Department of Health guidelines are being followed, e.g. the percentage of handwash basins in a facility with soap, water and towels available.
2. Process indicators: these rate how well individuals follow facility-based guidelines, but may also include how many individuals were trained on local IPC policy implementation, e.g. the percentage of hand hygiene compliance; the number attending training on tuberculosis (TB) infection control.
3. Outcome indicators: these measure the outcome that IPC programmes are trying to prevent, healthcare-associated infection, e.g. the facility’s infection rate from surgical site infections, urinary tract infections in catheterised patients and rates of antibiotic-resistant infections.

Structure and function of infection prevention and control programmes

1-8 What is the structure of infection prevention and control programmes at different levels?

The structure of IPC programmes varies from country to country. Many programmes have a central co-ordinating body situated within the national Department of Health or within the provincial (regional) healthcare administration. Each facility (both primary care clinics and hospitals) are then required to implement and adhere to the prescribed national or provincial IPC guidelines and policies. At individual facility level, the IPC programme should involve the facility management, the IPC committee and the IPC practitioner.

1-9 Who should take responsibility for the infection prevention and control programme?

Every healthcare worker has responsibility for preventing harm to themselves, fellow staff, visitors and patients. However, the final authority and responsibility for IPC lies with the facility management. They may delegate this role to the IPC team, but must ensure that the right support structures are in place to ensure a functional and effective IPC programme.

1-10 What are the minimum requirements for infection prevention and control programmes?

The minimum requirements for IPC programmes are the IPC standards that should be in place at all health facilities in the country. These provide protection and safety for patients and are based on the WHO recommended core components for implementing IPC.

1-11 What are the World Health Organisation's recommended core components for the implementation of infection prevention and control in health facilities?

The eight WHO core components are:

1. National IPC programmes: These must have clearly defined objectives and good practices to prevent healthcare associated infections (HAI) and address

antimicrobial resistance (AMR). A dedicated and trained team should be in place in each facility to implement these good practices.

2. IPC guidelines: Evidence-based IPC guidelines should be developed and implemented to achieve these good practices. Healthcare workers should be educated and trained to monitor the adherence of these guidelines to obtain successful implementation.
3. IPC education and training: The national IPC programme should support team and task-based strategies that include bedside and simulation training for all healthcare workers.
4. HAI surveillance: Facility-based surveillance should guide IPC interventions and detect outbreaks with feedback to the healthcare workers and national stakeholders.
5. Multilevel improvement strategies: National, provincial and district strategies should be co-ordinated and facilitated to improve good practices and reduce HAI and AMR at facility level.
6. Monitoring, evaluation and feedback: A national monitoring and evaluation programme of key performance indicators, such as hand hygiene, should determine whether IPC standards are met and activities performed. Relevant staff should receive regular feedback on the assessments.
7. Workload, staffing and bed occupancy: Bed occupancy should not exceed the facility's capacity and staffing levels in order to reduce the risk of HAI and spread of AMR.
8. Built environment, materials and equipment: At each facility materials and equipment for good hand hygiene must be available at the point of care. In addition, a clean and hygienic environment should be maintained. This must include all elements of water and sanitation health (WASH) infrastructure.

1-12 How should healthcare facilities implement improvements to their infection prevention and control programme?

A cycle of continuous improvement can be achieved by following these five steps:

Step 1: Prepare for action.

Step 2: Make a baseline assessment.

Step 3: Develop and execute an action plan.

Step 4: Evaluate the impact of the plan on the service.

Step 5: Sustain the programme over the long term.

Each step leads on to the next step in a circle.

1-13 How can healthcare facilities assess and measure improvements in an infection prevention and control programme?

Two national assessment tools are used:

1. **The National IPC Assessment Tool**: This standardised assessment tool is designed to determine what IPC core components are already in place and to identify gaps and weaknesses at the national level. The main purpose of the tool is to support implementation and provide a road map to guide IPC actions. It should be used for self-assessment by the national IPC team or external experts and consists of a scoring system to address the national core components.
2. **The Facility IPC Assessment Framework**: This assessment tool is designed to measure the IPC situation in health facilities. It aims to determine what core components are already in place and to identify gaps and weaknesses to guide action planning. It consists of a questionnaire and associated scoring system and should be used by healthcare professionals responsible for organising and implementing IPC measures at the facility.

1-14 What is an infection prevention and control team?

Ideally, an IPC team is made up of an IPC doctor and one or more IPC nurse practitioners, however, in some countries the IPC nurse practitioners function on their own. Preferably, an IPC doctor should be trained in infectious diseases, medical microbiology, public health or related specialities. The IPC nurse practitioner should be formally trained in IPC. The duties of the IPC team generally include:

- Performing and reporting on surveillance for healthcare-associated infection
- Investigating and advising on outbreak management
- Providing a clinical advisory service for infection prevention-related issues
- Revising and formulating policies
- Providing regular in-service training in IPC for all healthcare workers
- Auditing quality of care, high-risk procedures, and occupational injuries
- Reporting to and advocating for improved standards of care with facility management
- Liaising with all role players, e.g. laboratory, engineering, nursing, clinicians, sterile services, pharmacy, cleaning services, and the procurement division.

1-15 How many infection prevention and control nurse practitioners are needed?

Many countries provide guidance on how many IPC nurse practitioners (IPCNP) are required in order to deliver an effective IPC programme. This is reported as the beds per IPC nurse practitioner ratio. In well-resourced settings this is often one IPC nurse practitioner per 100 hospital beds, but in low-resource settings may be as low as one IPC nurse practitioner per 250 hospital beds. Owing to a lack of skilled staff, many countries may have only one IPC nurse practitioner per facility or per district. In low-resource settings, nursing staff in clinical units can be trained to fulfil some IPC practitioner functions (known as IPC link nurses).

1-16 What is an infection prevention and control committee?

An IPC committee is a multi-disciplinary group of healthcare facility staff who advise and assist with:

- Management of the IPC programme
- Policy development
- Procurement issues
- Patient safety
- Risk identification
- IPC training and education
- Antimicrobial and disinfectant use
- Surveillance of healthcare-associated infections.

The IPC committee usually consists of representatives from:

- Facility management
- Nursing management
- Clinicians
- The IPC practitioner or team
- Quality management
- Occupational health
- Pharmacy
- Cleaning services
- Sterile services
- Engineering department.

IPC committee meetings are usually held monthly or quarterly, with circulation of reports and meeting minutes to management and all other stakeholders in the facility.

1-17 What legislation supports infection prevention and control programmes?

Each country has its own legislation (laws) governing IPC. In case there aren't any local legislation available there are several international documents that provide recommendations for IPC programmes, including those available from the World Health Organization (WHO) and Centers for Disease Control (CDC), amongst others (see addendum).

Education of healthcare workers

1-18 Why should healthcare workers be educated about infection prevention and control?

In many countries, there is insufficient emphasis on IPC in the undergraduate training of medical, nursing and allied health professionals. In addition, the clinical training facilities and senior staff often provide poor examples of IPC best practice to students. New guidelines, equipment, procedures and even new diseases result in a need for regular updates to the healthcare workers' IPC knowledge. Education is also important to address workers' concerns, fears, stigmas and incorrect assumptions regarding transmission or prevention of healthcare-associated infections.

1-19 Which categories of healthcare workers should be trained in infection prevention and control?

All healthcare workers require at least a basic understanding of IPC principles. Since different categories of workers may have different information needs, it is recommended that IPC training sessions be tailored to the specific target audience, e.g. medical staff versus cleaning services staff. Critical information to include is training on standard and transmission-based precautions.

1-20 When and how often should healthcare workers be trained in infection prevention and control?

Ideally all new employees should receive induction (pre-employment) training in IPC. Annual refresher courses or short in-service training updates are recommended for all categories of healthcare staff.

1-21 Which education methods can be used to train staff in infection prevention and control?

The simplest and often most well-accepted format for training is face-to-face, small group teaching. This is, however, the most time-consuming teaching method, and may limit the number of staff that the IPC practitioner can educate. Incorporating short sessions into the weekly clinical schedule and utilising other staff for IPC education may be effective, e.g. using the sister-in-charge of a ward to give a demonstration on hand hygiene techniques at the morning ward handover rounds. Alternative methods include formal IPC courses, distance learning (including small-group, self-study and collaborative learning, video demonstrations and e-learning (online short courses).

1-22 Who should provide training of healthcare workers in infection prevention and control?

The responsibility of providing training in IPC usually falls to the IPC practitioner or IPC team. However, involvement of other senior healthcare workers is important as staff members are more likely to follow the advice of respected clinical leaders and colleagues. There may also be additional nursing staff appointed for clinical training at some facilities (called clinical co-ordinators). These clinical co-ordinators, as well as IPC nurses, may be able to provide some IPC training to their colleagues.

Audits in infection prevention and control

1-23 What is an infection prevention and control audit?

An audit is an assessment of practice based on pre-determined criteria. Audits are used as a quality management tool to improve patient safety and standards of care. In IPC, audits are used to monitor and evaluate how well a facility or clinical area is complying with specified standards of good IPC practice. Before an audit can be started, each facility must decide which standards or policies their performance will be measured against.

1-24 What is the purpose of infection prevention and control audits?

The purpose of performing an audit is to check how real-life observed practice in a facility or clinical area compares with accepted best-practice or standards of care. After the audit is completed, feedback with suggestions of how to improve practice is given to all stakeholders. This important step must not be forgotten. After the suggested changes or improvements have been implemented, the facility or clinical area should be re-audited. This process is similar to a quality improvement cycle.

1-25 Who should conduct infection prevention and control audits?

The people performing the audit should preferably be very experienced in the practices that they will be auditing. IPC practitioners are well-placed to perform such audits, but need to be impartial when evaluating practice in their own facility. For national audits, it is better to get auditors who do not work at the facility being assessed, as they are more likely to notice problems with practice than an IPC practitioner who works in the facility. All audits should have the approval and support of facility management. Staff at the facility or clinical area being assessed should be informed prior to performing the audit, but the auditor should attempt not to interrupt clinical work or influence clinical practice during the assessment.

1-26 What is needed to conduct an audit on infection prevention and control practice?

Several elements must be considered before starting with an audit:

- The reference standard against which the audit will be conducted must be accepted.
- Permission to conduct the audit must be obtained.
- An experienced auditor or audit team must be identified.
- The stakeholders to whom the report will be presented must be identified.
- An audit tool or questionnaire must be designed and approved.
- The scoring system for audit results must be decided on.
- Management should agree to a reasonable period of time for the audit recommendations to be implemented before requesting a re-audit of the facility or clinical area concerned.

1-27 How and to whom should the audit findings be reported?

The audit outcome should be presented to stakeholders both in written and oral format. The written document should provide clear and understandable feedback with itemised and prioritised recommendations for improvements. It is best to give suggested timelines and assign individuals responsible for the implementation of the audit recommendations, as this keeps the facility or clinical area managers accountable. The various practices audited can be divided into categories for ease of reading. Compliance should be evaluated as shown below. The final outcome of the audit may be reported as a percentage score or a symbol.

Table 1-1: An infection prevention and control audit tool

**Final score-sheet for IPC practice audit in Ward B, Hospital X**<br>**Date of audit: 13 March 2014**<br>**Auditors: Sister X and Doctor Y**			
**Practice category**	**Non-**	**Partially**	**Fully compliant (2)**

**Final score-sheet for IPC practice audit in Ward B, Hospital X**<br><br>**Date of audit: 13 March 2014**<br><br>**Auditors: Sister X and Doctor Y**			
	**compliant (0)**	**compliant (1)**	
**Environmental cleaning**			
1. There is a written policy for general cleaning of the ward			✓
2. Cleaning equipment is appropriately stored on the ward	✓		
3. All cleaning staff are trained in IPC			✓

**Hand hygiene**			
1. Running water is available at all hand-wash basins		✓	
2. Soap is available at all handwash basins		✓	

**Final score-sheet for IPC practice audit in Ward B, Hospital X**<br><br>**Date of audit: 13 March 2014**<br><br>**Auditors: Sister X and Doctor Y**			
3. Paper towels are available at all hand-wash basins	✓		

**Tally the scores**	**0**	**2**	**4**

Sub-total score	Actual score of 6 out of possible score of 12 = 50%

1-28 What infection prevention and control audit tools are available?

There are many well-designed tools available online for a variety of IPC audits, from comprehensive IPC programme audits, to hand hygiene, environmental cleaning, antibiotic usage and sterilisation and disinfection audits.

Policy development in infection prevention and control

1-29 What is a policy?

A policy is a document that records ‘a plan or a course of action intended to influence and determine decisions and action’. The purpose of a policy is to provide standard guidance on a particular topic, e.g the facility policy on needlestick injury (NSI) management, the facility policy on Covid- 19 isolation room usage.

1-30 When is a policy needed?

Circumstances and practices in healthcare settings change often. There are several reasons for drafting or revising a policy including:

- If no policy exists
- If a policy is outdated
- If there is a change in clinical practice, facilities, equipment or a new disease
- If legislation has changed or new laws have been introduced
- If there is a need to establish practice standards.

1-31 How are policies helpful in improving infection prevention and control?

From an IPC perspective, policies are useful for:

- Assisting healthcare workers to understand practices (as an educational tool)
- Supplying an agreed method of dealing with mishaps, e.g. needlestick injury or blood spills
- Ensuring healthcare workers are held accountable for their actions (since policies explain the minimum standard of care expected).

1-32 Who should be involved in drafting IPC policies?

Policies are usually drawn up by the IPC team, on behalf of a facility's IPC committee or facility management. It is essential that all role players are consulted when the policy is being drafted. This ensures that staff have a sense of ownership of the policy and increases the chance that they will actually follow the policy recommendations.

1-33 What is involved in the process of drafting IPC policies?

Drafting or revising a policy should follow a specified process to ensure that the policy will be both evidence-based and acceptable to the facility staff. The steps involved in policy development include:

- Decide who will be involved (the policy working group).
- Critically review any existing policy documents.
- Remove any elements that are no longer applicable or are not based on evidence.
- Add new elements as needed, e.g. if new evidence has emerged or a test method has changed.
- Share the draft policy widely and give stakeholders a deadline to respond.
- Modify the draft based on the suggestions and re-circulate for final changes.
- Present the policy to the IPC committee or facility management for approval.

1-34 Which components should be included in a policy?

Every policy requires certain components including:

- A title (stating the content and intention of the policy)
- A rationale (the reasons why the policy was written)
- A scope (the target audience or geographical area covered)
- The principles (refers to the laws/legislation, research evidence and infection control principles that the policy incorporates)
- A body (the actual recommendations or steps to be implemented)

- Dates (date of policy adoption and date that renewal is required – usually every two years)
- Contact information (include the names of the people who were involved in drafting the policy).

1-35 How should new IPC policies be communicated and implemented?

The process of communicating a new policy is much simpler if all stakeholders were consulted during the policy drafting process. Before launching the policy:

- Make sure the policy is written in simple, understandable language
- Ensure that all the resources are available to implement the policy
- Pilot or test the draft policy before wide implementation.

Once the policy is finalised and approved, send out the final policy to all role players with a stated date for policy implementation. Call a meeting to go through the policy with all relevant role players.

Arrange for staff to be trained on the policy. Monitor the outcome of the new policy, providing opportunities for staff to report difficulties or request support for the implementation process. Make sure the policy is included in the infection control manual and that the document is easily accessible to staff.

1-36 What is a standard operating procedure (SOP)?

A standard operating procedure (SOP) is a written explanation of how to perform a practical task, e.g. how to clean a laryngoscope after use. SOPs are written using verbs or action words to describe the process, e.g. put on gloves and an apron, disassemble the laryngoscope, unscrew the light bulb, scrub the blade with soap and water. The instructions and steps should be very specific so that there is no uncertainty or confusion. The draft SOP should be produced after wide consultation with stakeholders. Once finalised and approved, the SOP

should be prominently displayed in the appropriate place, e.g. hand hygiene posters at wash basins; urine testing SOP in sluice rooms.

1-37 When is a standard operating procedure needed?

Most SOPs are written for high-risk tasks where the potential negative consequences for an incorrect practice are serious. SOPs can also be used to simplify complicated processes by breaking a task down into steps. SOPs are also useful for induction training of new staff members.

1-38 What is a guideline?

Guidelines are usually written to provide standard recommendations for:

- the treatment of a clinical condition, e.g. guidelines for the treatment of syphilis
- management of a particular circumstance, e.g. guideline for the management of needlestick injuries.

The format resembles that of a policy or SOP.

Report writing in infection prevention and control

1-39 What is an infection prevention and control report?

Reports in IPC are used to document findings and facts about a particular situation (e.g. outbreaks), services (e.g. the IPC programme) or practices (e.g. hand hygiene compliance). The purpose of an IPC report is to share factual information and to provide recommendations for improvement.

1-40 What should be included in the report?

An IPC report may be simply written but should provide sufficient detail (both written and in graphs/figures) to allow the reader to understand the content. The following components are included in most reports:

- Summary (a concise version of the report)
- Background (why the investigation or audit was undertaken)
- Aims (what should be accomplished)
- Methods (the steps used in the investigation or the audit checklist/tool used to measure compliance)
- Findings (what was observed or discovered, reported as numbers and percentages, or statistics)
- Discussion (includes the recommendations for changes required to improve IPC practice or avoid further outbreaks)
- Date of the report and the names of parties involved in drafting the report.

Report: Investigation of an outbreak of Klebsiella pneumoniae sepsis on the Neonatal Unit

Summary

In January 2014, eight neonates were diagnosed with *Klebsiella pneumoniae* bloodstream infection. Molecular testing showed that the infections were related. Investigation of the outbreak revealed the source to be inadequately decontaminated ventilator tubing. Hospital management agreed to stop the practice of recycling used ventilator tubing. No further cases of Klebsiella pneumoniae sepsis have been reported to date.

Background

Klebsiella pneumoniae is a common neonatal pathogen which can cause bloodstream infections, pneumonia and meningitis. Outbreaks of *Klebsiella pneumoniae* sepsis in neonatal units are not infrequent. Poor hand hygiene, contaminated medication or equipment and inadequate environmental cleaning may be responsible for outbreaks with this pathogen.

Aims

To identify the cause of the outbreak.

Methods

Following identification of eight neonates with *Klebsiella pneumoniae* sepsis in the neonatal unit over four weeks an outbreak investigation was undertaken. The following methods were used:

- A line list and Gannt chart were drawn up
- Audits of hand hygiene compliance and environmental cleaning were conducted in the neonatal unit
- Microbiological cultures (swabs) from equipment, surfaces and ventilator tubing were taken
- Molecular analysis techniques (PCR sequencing) were used to determine if the bacterial isolates were related.

Findings

The line list revealed that ventilation was a common risk factor for all eight affected neonates.

Overall levels of hand hygiene were low (average 30%, 125 observations).

Environmental cleaning levels were acceptable (audit score 24/30 = 80% compliance).

Strain typing of the eight neonates and the six isolates from the ventilator tubing revealed a closely related strain, implying that the inadequately decontaminated tubing was the source of this outbreak. On inspecting the washer disinfector used to clean the ventilator tubing in the sterile services department, it was found to be outdated with a faulty temperature gauge.

Recommendations

1. The practice of recycling single-use items should be stopped with immediate effect.
2. The outdated washer disinfector in the sterile services department should be replaced.
3. Sterile services department staff should be re-trained on validation of decontamination.
4. Hand hygiene compliance in the neonatal unit should be reinforced.

Date of report: 21 February 2014

Investigation Team: Sr N Khumalo (IPC practitioner), Dr M Smith (Microbiology), Dr G Sithole (Neonatal Unit)

Occupational health and safety programmes

1-41 What is an occupational health and safety (OHS) programme?

OHS programmes aim to promote and protect the health and safety of all healthcare workers.

In general the OHS programme should perform:

- Pre-employment screening
- Workplace safety and risk assessments
- Surveillance and medical evaluations for occupational injuries and diseases
- Immunisation for occupationally acquired diseases.

1-42 What is the link between infection prevention and control (IPC) and occupational health and safety (OHS) programmes?

The IPC and OHS services at a facility should have a close working relationship, ensuring the safety of patients, visitors (IPC) and staff (OHS and IPC). In some low-resource settings, the responsibility for IPC and OHS are combined in a single IPC/OHS practitioner post. IPC and OHS work together to monitor for and prevent transmission of hazardous biological agents, e.g. tuberculosis (TB), blood-borne diseases (HIV, hepatitis B and C), among others.

1-43 Which diseases and activities of the occupational health and safety programme have relevance to infection prevention and control?

Particular programmes where IPC and OHS services should combine their efforts are:

- Needlestick injury monitoring and prevention programmes
- Tuberculosis occupational disease monitoring and prevention programmes
- Immunisation of healthcare workers, e.g. hepatitis B and influenza
- Policy and guideline development with relevance to IPC and OHS
- Education and training of healthcare workers about occupationally acquired diseases and injuries.

1-44 How should needle-stick injuries be handled?

Every healthcare facility should have a needlestick injury (NSI) policy that is familiar and accessible to all staff and is regularly updated (at least every two years). Training on the NSI policy should be mandatory for all healthcare workers at pre-employment training or staff induction. The general procedures and principles that should be addressed in a NSI policy include:

- Perform first aid (remove the sharp, wash the affected area with soap and running warm water, do not suck the wound).
- For eye or mouth splashes, rinse out with lots of water (use eye washout kits if available).
- Report the injury to a supervisor and to the occupational health service immediately.
- Establish (where possible) the HIV, hepatitis B and C status of the source case.
- Establish the injured healthcare worker’s immunity to hepatitis B.
- Establish the injured healthcare worker’s HIV status and if HIV-positive, establish whether their CD4 and HIV viral loads have been checked within the last six months.
- Provide post-exposure prophylaxis for HIV as soon as possible (preferably within 1–2 hours of the injury) and if necessary for hepatitis B.
- Provide pre- and post-test counselling for the source patient and injured healthcare worker.
- Provide regular follow-up, support and testing for HIV and hepatitis sero-conversion to healthcare workers exposed to blood-borne viruses.

1-45 How can needlestick injuries be prevented?

- Healthcare workers should be educated never to recap used needles.
- Used needles should be immediately discarded in a puncture-proof sharps box.
- Sharps boxes should be located within arm’s reach of a procedure.

- Sharps boxes must be replaced when more than two-thirds full.

Case Studies

Case study 1

A new 1000-bed hospital is being built in a community with a high burden of tuberculosis (TB) and HIV. The hospital manager is busy recruiting staff to run the infection prevention and control programme.

1. What should the main goals of the IPC programme be?

The primary goals of the new hospital's IPC programme should be:

To prevent susceptible patients acquiring pathogenic (disease-causing) micro-organisms

To contain the spread of antimicrobial resistant infections.

2. How many IPC practitioners will the new hospital need?

Since this will be a large 1000-bed hospital with a heavy burden of infectious diseases, the hospital manager should allocate sufficient human resources to IPC. Ideally he should appoint an IPC team, including an IPC doctor and at least four IPC nurse practitioners (that is one for every 250 beds).

3. What activities should the hospital manager add to the job description of the IPC practitioners?

The daily duties of the IPC practitioners would include:

Organising surveillance for healthcare-associated infections

Providing advice and leadership in outbreak investigation

Developing and delivering training on IPC to healthcare workers

Developing and implementing IPC-related policies and procedures

Auditing the quality and effectiveness of healthcare facility environmental cleaning

Auditing the quality and effectiveness of disinfection and sterilisation practices

Implementing local, national or international best-practice guidelines for prevention of infection transmission in clinical care.

4. What other structure should the hospital manager create to assist the IPC team?

The manager should ensure that an IPC committee is formed as soon as possible after the hospital opens. An IPC committee is a multi-disciplinary group of healthcare facility staff who volunteer or are elected to advise and assist with management of the IPC programme. The manager should ensure that IPC committee meetings are held regularly with circulation of reports and meeting minutes to management and all other stakeholders in the facility.

Case study 2

A newly appointed IPC practitioner notices that staff at her clinic have limited understanding of and poor implementation of airborne isolation precautions for prevention of tuberculosis transmission. On questioning different categories of staff, she realises that they have had very little IPC education during their training and none at all since they started working at the clinic.

1. Why do these healthcare workers need training in IPC?

In many countries, there is insufficient undergraduate training in IPC for medical, nursing and allied health professionals. In addition, once they enter the workplace, healthcare workers often follow the poor or incorrect practices of senior colleagues. Over time new guidelines, equipment, procedures and even new diseases may arise, resulting in a need for regular in-service IPC training for healthcare workers.

2. Who should the IPC practitioner train?

All healthcare workers require at least a basic understanding of IPC principles. Since different categories of workers may have different information needs, it is recommended that IPC training sessions be tailored to the specific target audience. In the case of training on isolation precautions for Covid-19, every staff member who comes into contact with patients will need training, including cleaners, porters, nurses, doctors, radiographers and clinic reception staff.

3. When should staff members receive IPC training?

Ideally all new employees should receive induction (pre-employment) training in IPC. Annual refresher courses or short in-service training updates are recommended. In this case, the IPC practitioner may need to prioritise who is most at risk and then start training for this group first.

Case study 3

The facility manager requests the IPC practitioner to provide copies of all policies developed, audits performed and reports written in the last year. The IPC practitioner has to explain to the manager's secretary which documents to file under policies, audits and reports.

1. What is a policy and what are some examples of documents that could be included?

A policy is a document that provides standard guidance on a particular topic. The IPC practitioner submitted a new policy and several policies that were updated in the last year, including:

- The facility policy on covid-19 infection prevention and control (covid-19-IPC)
- The facility policy on needlestick injury (NSI) management
- The facility policy on isolation room usage.

2. What is an audit and what are some examples of audit reports that could be included?

An IPC audit is an activity performed to evaluate how well a facility or clinical area is complying with IPC standards. Examples of IPC audit reports that could be supplied to management include:

- Annual facility-wide audits of hand hygiene compliance
- Audits of compliance with healthcare waste management
- Audits of environmental cleaning.

3. What is an IPC report and what are some examples of reports that could be included?

Reports in IPC are used to document findings and facts about a particular situation (e.g. outbreaks), services (e.g. the IPC programme) or practices (e.g. hand hygiene compliance). The IPC practitioner could submit reports on the following:

- Outcome of outbreak investigations
- Reports of healthcare-associated infection rates

- Reports on training and education of staff in IPC.

Case study 4

A nurse is carrying a sharps container and the lid which was loosely placed on top of the container falls off. All the sharps fall out and one needle and syringe which appears unused jabs him on the lower leg. The sharps container had been placed next to a patient who was having a fingerprick glucose test taken.

1. What steps should he take immediately after the accident?

The nurse should call his superior and report the incident immediately. She should then report to the occupational health department or officer, with the needle and syringe that caused the accident. The spillage should be cleared up by an experienced person in full protective equipment and with the appropriate brush and pan.

2. What should the occupational health officers do for him as a staff member?

Check his immunisation record and immune status. Take blood from the porter and from the source patient for HIV, hepatitis B and C and send for testing. If his immunisation is inadequate, fast track hepatitis B immunisation and start on HIV post-exposure prophylaxis (PEP) within two hours. If the source blood is HIV-negative then the PEP can be stopped. If the member of staff is HIV positive then his CD4 count and viral load should be checked.

3. What is the expected risk from this accidental needle stick injury?

This needle and syringe did not appear to have blood in it therefore the risk is less than if there had been blood in the needle and the barrel of the syringe. Nonetheless, it is essential that full precautions as described above be taken.

Adapted from:

- Infection Prevention and Control Paperback – August 23, 2020 edition by Dr Angela Dramowski (Author), Prof David Woods (Author), Prof Shaheen Mehtar (Author)

- World Health Organization (WHO): www.who.int

- Centers for Disease Control and Prevention (CDC): www.cdc.gov
