# Supplementary material for: WhatsApp-Delivered Intervention for Continued Learning for Nurses in Pakistan During the COVID-19 Pandemic: Results of a Randomized-Controlled Trial
Source: Front Public Health. 2022 Feb 15;10:739761. doi: 10.3389/fpubh.2022.739761 (PMC8885589; doi:10.3389/fpubh.2022.739761)
Supplement: Supplementary file 2 [file Data_Sheet_2.pdf]

<u>Intervention Training Material for Nurse Leadership Study</u>

Module 2: Infection Prevention and control - COVID-19

Contents

Intervention Training Material for Nurse Leadership Study 1
Module 2: Infection Prevention and control - COVID-19 1
Objectives 4
Introduction to COVID-19 5
1-1 What is COVID-19? 5
1-2 What is a coronavirus? 5
1-3 Where and when did COVID-19 first appear? 6
1-4 How is coronavirus spread between people? 7
1-5 Can people who are clinically well spread coronavirus? 8
1-6 What is the incubation period for COVID-19? 8
1-7 How many people does one person usually infect with coronavirus? 9
1-8 How many people with coronavirus infection become ill? 9
1-9 How many people with COVID-19 will become seriously ill? 9
1-10 Which people are at increased risk of becoming seriously ill with COVID-19? 10
1-11 How many people with COVID-19 are expected to die? 11
1-12 What is the COVID-19 pandemic? 11
1-13 Can COVID-19 be cured? 12
1-14 How can the clinical diagnosis of COVID-19 be confirmed? 12
1-15 How can people who have had COVID-19 be identified? 13
1-16 Can people who have recovered from COVID-19 become infected again? 13
How to prevent the spread of COVID-19 14
1-17 Is there a vaccine for COVID-19? 14
1-18 What preventative measures can be taken by the general public to stop the spread of COVID-19? 14
1-19 What is thorough handwashing? 15
1-20 What is respiratory hygiene? 15
1-21 What is physical distancing? 16
1-22 Is it helpful to wear a simple cloth face mask and gloves when going out in public? 16
Video on Mask Safety: 17

1-23 How effective is staying at home? 17
1-24 What can national governments do to slow the spread of COVID-19? 18
1-25 What does it mean to 'flatten the curve'? 19
1-26 What is a community lockdown? 19
1-27 What are the social consequences of community lockdown? 20
1-28 What are the challenges of locking down poor communities? 21
1-29 What is contact tracing? 21
Clinical features of COVID-19 22
1-30 How does COVID-19 present? 22
1-31 What are the features of mild COVID-19? 22
10-32 What is the most important complication of COVID-19? 23
Management of people with suspected coronavirus infection 23
1-33 What is the management of asymptomatic people with suspected coronavirus infection? 23
1-34 What should a patient with suspected COVID-19 do when they go to a health facility? . 24
1-35 How are patients screened for COVID-19? 24
1-36 Who should be screened? 25
1-37 How should patients with suspected COVID-19 be triaged? 26
Management of patients with mild COVID-19 26
1-38 What is the management of patients with mild COVID-19? 26
1-39 How should patients with mild COVID-19 self-monitor? 27
1-40 Can patients with COVID-19 still infect others after their symptoms disappear? 27
Management of patients with moderate or severe COVID-19 27
1-41 What is the hospital management of patients with moderate or severe COVID-19? 27
Protecting healthcare workers 29
1-42 What can healthcare workers do to protect themselves? 29
1-43 What is personal protective equipment (PPE)? 29
1-44 Can personal protective equipment be reused? 30
1-45 For which procedures are N95 respirators needed? 31
1-46 Can one reuse a N95 respirator? 31
1-47 How should you handle your uniform or work clothes? 32
1-48 How should infectious waste be handled and disposed of? 32
1-49 How should contaminated linen be handled? 32
1-50 What cleaning of surfaces and equipment is required in COVID-19 areas? 32
1-51 What protection should community health workers use? 33

1-52 What is a screening and testing triage area outside a hospital? 33
1-53 How can coronavirus spread be limited in hospitals? 33
Case study 1 34
1. Why should the doctor worry that he may have COVID-19? 34
2. Does he have any factors which place him at high risk of severe infection? 35
3. Should he have gone to the doctor? 35
4. How could the diagnosis of COVID-19 be confirmed? 35
5. How severe is this patient’s illness? 35
6. How should he be managed? 35
Case study 2 35
1. How long will she need to wait if she is to develop symptoms of COVID-19? 36
2. Is it safe for her 10-year-old daughter to visit her if they do not touch or kiss? 36
3. If she went to a coronavirus screening centre should she wear a mask? 36
4. When will she be feeling better if she has mild COVID-19? 36
5. Would influenza immunisation have protected her from COVID-19? 36
Case study 3 37
1. What is personal protective equipment? 37
2. Is a surgical mask adequate? 37
3. What is a N95 respirator? 37
4. Can a N95 respirator be reused? 37
5. What should be used to disinfect hospital equipment? 38
6. What advice should be given about removing work clothes? 38
Adapted from 38
1. Book - Infection Prevention and Control Paperback – August 23, 2020 edition by Dr Angela Dramowski (Author), Prof David Woods (Author), Prof Shaheen Mehtar (Author) 38

The objectives of this module include

- Understanding the origin and terminology for COVID-19
- Describing how the coronavirus is spread
- Understanding how to limit the spread in communities
- Describing the clinical presentation and progression of the disease
- Understanding the principles of managing people with COVID-19
- Protecting healthcare workers from COVID-19.
- Preventing COVID-19 spread in hospitals.

Introduction to COVID-19

1-1 What is COVID-19?

COVID-19 is an acute illness caused by a new (novel) coronavirus which was first identified in 2019. COVID-19 stands for Coronavirus disease of 2019. The new coronavirus which causes COVID-19 has been named Severe Acute Respiratory Syndrome Coronavirus 2 or SARS-CoV-2. Humans have never been exposed to this new virus before and therefore have no immunity to it.

NOTE

> The virus causing COVID-19 was named SARS-CoV-2 as it is related to a previous coronavirus, SARS-CoV, which caused multi-country outbreaks of a different severe acute respiratory syndrome in 2002.

1-2 What is a coronavirus?

Coronaviruses are a large group of viruses which commonly cause disease in both animals and humans. In humans coronaviruses usually cause mild upper respiratory tract illnesses such as the common cold or a sore throat. However, sometimes a new coronavirus appears which may cause severe illness or even death.

Coronaviruses sometimes cross over from an animal species to infect humans. This is believed to be the origin of new human coronaviruses such as SARS-CoV-2.

Here onwards SARS-CoV-2 will be referred to as coronavirus.

NOTE

SARS-CoV-2 is an enveloped single strand RNA virus which needs to enter a living animal cell in order to multiply. When viewed under an electron-microscope the virus looks like a crown with many spikes, hence the name coronavirus. The spike proteins attach to specific receptor sites on the host cell. The viral RNA then enters the mucosal cells of the human hosts' upper airways, where it multiplies and spreads to other parts of the body.

1-3 Where and when did COVID-19 first appear?

In a food market in Wuhan, the capital city of Hubei province in central China during December 2019. In the market a wide range of both dead and live animals from all over the world were sold. It is believed that the COVID-19 virus crossed from a bat (the primary animal reservoir) to a pangolin (the intermediate host) and finally to a human.

The ability to change from an animal infection (zoonosis) to a human infection is due to a change (mutation) in the genetic code of the virus. This is more likely when animals, often under very unhygienic conditions, and humans are in close contact with one another. Other viruses, such as the influenza virus, have also crossed over from one host, such as a bird or pig, to humans.

NOTE

Twice before in the last 20 years there have been serious epidemics caused by a coronavirus:

1. In 2002 a coronavirus (SARS-CoV) caused severe acute respiratory syndrome (SARS). The infection started in the Chinese province of Guangdong and affected 8000 patients of which 10% died.

2. In 2012 another coronavirus (MERS-CoV) caused Middle East respiratory syndrome (MERS). The infection started in Saudi Arabia and affected 2500 patients of which 34% died.

1-4 How is coronavirus spread between people?

There are a number of ways that coronavirus can be spread:

- **Droplet spread**: The main method of spread from one person to another is by respiratory droplets produced when an infected person coughs or sneezes. Shouting, singing or even speaking may also spread the virus. The droplets of saliva or nasal secretions in the air contain coronavirus and may land in another person's mouth, eyes or be breathed in. The closer the people are to each other the greater the risk of spread. These droplets usually settle to the ground or land on surfaces such as table tops and chairs within seconds or minutes.
- **Contact spread:** If a person coughs or sneezes into their hand and then touches a surface such as a tap, door handle or phone, coronavirus may be transferred to someone else's hand if they touch the same surface. If the second person then touches their face (mouth, nose or eyes) coronavirus on their hand may infect their mucous membranes. Coronavirus may survive on a solid surface for 24 hours or longer if not removed by cleaning or disinfection. People may not even see who infects the contaminated surfaces.
- **Airborne spread:** New evidence has emerged that some coronavirus may spread by aerosols (tiny virus-containing particles that float in the air and may be breathed in). Airborne transmission of SARS-CoV-2 can occur during medical procedures that

generate aerosols ("aerosol generating procedures"). WHO, together with the scientific community, has been actively discussing and evaluating whether SARS-CoV-2 may also spread through aerosols in the absence of aerosol generating procedures, particularly in indoor settings with poor ventilation.

- **Faeco-oral spread:** Coronavirus may also be present in the stools of infected people. Although it is not currently known how important this route of infection may be it could also be a method of viral spread.

1-5 Can people who are clinically well spread coronavirus?

Yes. The risk of spread is greatest when a person has symptoms of infection, such as a cough, although spread can also occur from people who have an asymptomatic infection ('silent spreaders'). Therefore, all people with coronavirus infection can infect others. Research has shown that for a few days before the onset of symptoms and even after clinical recovery (resolution of symptoms), infected people may shed coronavirus from nasal secretions and in stools. The duration of coronavirus shedding during and after infection is still uncertain, but probably lasts between 7 to 14 days in most people.

NOTE

The greater the viral load, the greater the risk of coronavirus spread. Some 'super-spreaders' probably have a high viral load and therefore are very infectious.

1-6 What is the incubation period for COVID-19?

About 5 days but this may range from 4 to 14 days. The incubation period is the time between exposure to coronavirus and the first symptoms of illness. People can spread the coronavirus to

others during the incubation period, before symptoms of infection appear. This is known as pre-symptomatic transmission.

1-7 How many people does one person usually infect with coronavirus?

On average an infected person will spread the virus to 3 other people if protective measures are not taken. This is 3 times more than the influenza virus. Therefore, the infectiousness (‘spreadability’) of coronavirus is greater than the influenza virus. However, it is far less infectious than some viral diseases such as measles.

NOTE

> ‘R_0’ is the reproductive ratio of a new virus or the number of people that an infected person is likely to infect. COVID-19 has a R_0 of 3. The higher the R_0 of a virus the more infectious it is. The aim of population management is to reduce the R_0 to less than one, so that the viral transmission chain is interrupted.

1-8 How many people with coronavirus infection become ill?

It is believed that about 70% of infected people will become ill while about 30% (possibly many more) will have no symptoms (asymptomatic). It is common for children to have an asymptomatic infection. With widespread testing this very important information will become available.

1-9 How many people with COVID-19 will become seriously ill?

Currently it seems that about 80% of symptomatic patients with laboratory-proven coronavirus infection will have a mild, self-limiting illness and recover fully. About 15% will have a moderate illness and need hospitalisation for oxygen and other supportive therapy. A further 5% will

become critically-ill and need intensive care, usually requiring assisted ventilation. Over time more accurate estimates from different countries will become available. About 20% of symptomatic patients with COVID-19 will need hospitalisation.

1-10 Which people are at increased risk of becoming seriously ill with COVID-19?

People at increased risk for serious illness are:

- Older people, especially those over 70 years of age. The older they are the greater the risk as the immune system weakens in the elderly.
- People with chronic lung conditions such as emphysema or tuberculosis and other conditions (co-morbidity) such as heart disease, vascular disease, cancer, hypertension, diabetes and obesity. The effect of COVID-19 on people living with HIV infection is as yet not known, but may increase the risk for severe disease, as is seen for influenza. However, people with an undetectable viral load on antiretroviral treatment may not be at increased risk.
- Healthcare workers who care for many infected patients and are exposed to a greater infective dose of the virus.

Children and young people infected with coronavirus usually do not become seriously ill, although some deaths in younger individuals have occurred. They may also be at lower risk of getting infected and passing on coronavirus. However children and young people may infect older members of their family.

The evidence for in-utero transmission of coronavirus from mother to fetus is very limited, but there have been several instances of respiratory transmission from infected mothers to their

newborn babies. However, the disease has been mild with few deaths reported. Breastfeeding is safe but an infected mother should wear a mask when feeding her baby and wash her hands carefully before handling her baby.

1-11 How many people with COVID-19 are expected to die?

The percentage of people who will die from COVID-19 (the case fatality ratio) remains uncertain as many people with asymptomatic infections are not detected and counted. Present data from high-income countries suggests about 3% of people with clinical infection will die despite current treatment. By comparison, the mortality rate with seasonal influenza is less than 1%.

With widespread screening to detect asymptomatic cases the mortality rate for all people infected with coronavirus is expected to be less than 3%. However, the mortality rate in poor countries may be higher.

NOTE

> Only when detailed surveys of infection rates in different communities are done will the true mortality rate of COVID-19 be known as the number of asymptomatic cases in different age groups and social classes remains uncertain. The number of deaths due to an undiagnosed coronavirus infection outside a health facility is also unknown.

1-12 What is the COVID-19 pandemic?

The coronavirus infection first spread locally in China but soon appeared in most other countries of the world. In March 2020 the World Health Organization (WHO) declared the infection a

pandemic. In contrast to an epidemic, which is the local rapid spread of an illness, a pandemic is where the illness is spread to many countries.

1-13 Can COVID-19 be cured?

Currently there is no medication proven to kill the coronavirus and cure COVID-19. However, many clinical drug trials show great promise and are being evaluated as potential treatments.

NOTE

> The spike proteins of coronavirus attach to ACE2 receptors on the surface of type 2 pneumocytes of the lung which allows the RNA of the virus to enter the cell. Viral proteins and RNA are then produced in the cell to create large numbers of new virus. It is hoped that new treatments will interfere with the attachment and reproduction of coronavirus. The use of commercially produced anti-viral antibodies (coronavirus immunoglobulin) or convalescent serum of people who have recovered from COVID-19 may help patients with severe illness.

1-14 How can the clinical diagnosis of COVID-19 be confirmed?

By identifying coronavirus in the upper respiratory tract. This is done by taking a nasopharyngeal or oropharyngeal swab and sending it to a laboratory to identify the presence of the coronavirus. This test is generally reliable in people who are clinically ill but may not detect coronavirus in about 15% of truly infected people (a false negative test), especially those with asymptomatic infection.

Efforts are underway to develop and validate a variety of rapid, bedside tests to screen for the virus or for antibodies to the virus, which will greatly improve the diagnosis of coronavirus infection.

NOTE

> A number of molecular tests, such as polymerase chain reaction (PCR) tests, are being used to identify the SARS-CoV-2 virus.

1-15 How can people who have had COVID-19 be identified?

People who have had either symptomatic or asymptomatic COVID-19 will have antibodies (IgM and later IgG) to the SARS-CoV-2 virus. A blood test to identify these antibodies will allow for assessing the scope of the pandemic and identifying individuals who are now immune to the infection and can return to their normal activities. Soon a bedside test on a drop of blood will be available for both health facility and home testing.

1-16 Can people who have recovered from COVID-19 become infected again?

Probably not as they should be protected by antibodies. However, if the coronavirus develops new mutations, re-infection with a slightly modified strain is theoretically possible as similar changes (known as antigenic drift) occur for the influenza virus each year, necessitating annual immunisation.

How to prevent the spread of COVID-19

1-17 Is there a vaccine for COVID-19?

In November three vaccines have been announced, which claim to be more than 90 per cent effective based on initial trial results. In addition to this there is a huge international effort to produce more effective vaccines of different types. Only when an effective vaccine is administered to large proportions of a country's population, will immunisation be able to control and finally halt the spread of COVID-19.

1-18 What preventative measures can be taken by the general public to stop the spread of COVID-19?

There are a number of simple steps that can be taken to reduce the risk of coronavirus spread:

- Frequent and thorough handwashing for minimum of 20 seconds with soap and running water
- An alcohol-based hand rub or hand spray (hand sanitiser)
- Covering the mouth with a flexed elbow or tissue when coughing or sneezing (respiratory hygiene)
- Avoid touching your face with unwashed hands
- Clean frequently touched surfaces such as door handles, phones and computer keyboards
- Keeping 1 to 2 metres from other people (social distancing)
- Stay at home.

- A nationwide information and education campaign.
- Wearing a face covering such as a home-made cloth mask.
- Avoid meeting people indoors if possible.
- Improve ventilation by keeping windows open.

1-19 What is thorough handwashing?

Regularly washing both hands well with soap and running water for 20 seconds and thorough hand drying. Make sure that all surfaces of the hands, fingers and thumbs are washed. Do not wash in a basin. Hands should be thoroughly dried with a paper towel after washing. Switch off the tap with the used paper towel.

A 60 to 70% alcohol-based hand rub or spray can also be used, particularly if soap and water are not available.

In the home, school and general workplace environment, hands should be washed before preparing food, after using the toilet and after handling tissues or respiratory secretions. Good handwashing is an important way to avoid coronavirus infection.

In the healthcare setting, the five moments for hand hygiene should be followed: before touching a patient, before clean (aseptic) procedures, after body fluid exposure, after touching a patient, and after touching patient surroundings.

1-20 What is respiratory hygiene?

This means turning your head away from others and covering your mouth and nose with your bent elbow or a paper tissue when you cough or sneeze. Dispose of the used tissue in a bin immediately. Never spit in public. It is the responsibility of everyone to protect others around them as well as

protecting themselves. Good handwashing after coughing and handling respiratory secretions (used tissues) is another important way to avoid coronavirus infection.

Always remember to ‘keep your droplets to yourself.’

1-21 What is physical distancing?

Getting no closer than 1 metre, and preferably 2 metres, away from another person, especially if they are coughing or sneezing. No shaking hands, touching, hugging or kissing. Physical (social) distancing is very important when doing essential shopping or standing in queues or having contact with other people indoors. While physical distancing is important, social contact via phone or the internet is also important to keep emotionally well.

A close contact is spending more than 15 minutes indoors with an infected person when you are both not wearing face masks and being less than 1 metre apart.

A casual contact is being near an infected person but when the above criteria are not met. The risk of transmitting coronavirus is much greater with close contact than with a casual contact.

1-22 Is it helpful to wear a simple cloth face mask and gloves when going out in public?

Wearing a simple paper or cloth mask in public will help protect others if you have coronavirus infection and also help protect you from the droplet spread of coronavirus by others. Gloves may get contaminated from infected surfaces and can then spread the coronavirus if you touch your face. Wearing a face mask may help you remember not to touch your face.

In a crowd, such as in a taxis, food stores or at a funeral, it would help if everyone wears a simple cloth mask as this could reduce the risk of an asymptomatic person shedding virus into the air. Cloth masks should be washed with soap and hot water and ironed after use. Wash hands after removing a mask. It is best if everyone has two cloth masks. The public should not use surgical masks or N95 respirators as these are in short supply for health workers.

Video on Mask Safety:

https://www.youtube.com/watch?v=adB8RW4I3o4

1-23 How effective is staying at home?

Staying at home means restricting the movement of a healthy person or families by getting them to stay at home. This is probably the most effective way of protecting yourself and your family from infection. Staying at home stops the coronavirus from spreading through contamination of the air and surfaces. People staying at home should have access to good information on COVID-19.

‘If in doubt don’t go out.’

Compulsory self-isolation is used when a person who is infected or suspected of being infected with coronavirus has to stay at home and does not mix with other people in order not to spread their infection. Quarantining usually means that someone who is well but has recently been in close contact with an infected person stays away from other people for 10 days when they may be in the incubation period for coronavirus.

1-24 What can national governments do to slow the spread of COVID-19?

There a number of steps that many governments are taking:

- Screening to prevent potentially infected people entering the country
- Testing anyone with suspected coronavirus infection
- Nationwide screening to identify people infected with coronavirus who have mild symptoms (active surveillance)
- Tracing people who have been in contact with someone who has COVID-19 (contact tracing)
- Isolating people with coronavirus infection or suspected of having coronavirus infection
- Quarantining people who are not infected but have been in close contact with an infected person or have arrived from another country with high rate of infection
- National regulations to prevent the public mixing with other people (community lockdown)
- Providing information via the media on how to avoid infection
- Giving the public accurate information about the progress of the pandemic
- Promoting the use of masks in public.

These efforts are all aimed at ‘flattening the curve’.

With active surveillance large numbers of the population are screened for coronavirus. This makes it possible to get a true idea of how many people are infected and what percentage are asymptomatic. An accurate mortality rate for infected people can then be calculated. It is vitally

important for projecting the course of the pandemic and planning for the care of ill patients. It also helps manage the impact on society and the economy.

1-25 What does it mean to 'flatten the curve'?

Without intervention COVID-19 spreads rapidly through a community with the number of infected people doubling every 2 to 3 days. This results in a steep peak in the number of infected people which places an enormous strain on health services, especially intensive care units. The aim is to reduce the speed at which new people become infected and so decrease the daily rate of infections and 'flatten the incidence curve'. This approach aims to reduce the total number of patients needing healthcare at any one time (mitigate the epidemic), by spreading the infections out over a longer time period to allow health facilities to better manage the numbers of severely ill patients. The capacity of the health system to manage cases of COVID-19 will be exceeded if the curve is not flattened. The best method to flatten the curve is community lockdown to reduce opportunities for coronavirus transmission.

1-26 What is a community lockdown?

With community lockdown everyone has to remain at home, not mix with others and not move around. This policy of enforced isolation aims to slow the spread of coronavirus and thereby save lives. This is a drastic action as it interferes with economic activity and the civil rights of citizens. However, it is the only way of reducing the number of infected individuals until a vaccine against COVID-19 becomes available to all.

With community lockdown most companies, shops, schools, places of higher learning, meetings, churches, sports clubs and any space where people congregate must be closed. Exceptions include health facilities, family practitioners, a limited number of food stores, banks, fuel stations, and other essential services. This causes severe social and economic disruption, especially for the poor.

With community lockdown (containment) there is a greater chance that public health tracing teams can identify infected individuals, trace and screen their contacts and ensure isolation of all infected individuals at home or hospital. This task is expensive and requires many health personnel. However, only by preventing the spread of the virus and tracing contacts can the rapid transmission of coronavirus in a community or population be avoided. Failure to do this would be a catastrophe with large numbers of citizens, especially the elderly, dying.

NOTE

> The aim of containment or community lockdown is to reduce the reproductive ratio (R_0), i.e. the number of people that an infected person is likely to infect.

1-27 What are the social consequences of community lockdown?

Being isolated is not natural and causes stress in many people. They may feel sad, scared, angry or very lonely. It is important to keep in contact with family or friends by email or phone. Do not obsess over television news reports all day and make sure that you only get scientifically correct information. It is important to establish a routine, get up and get dressed in the morning, have a good diet, get enough sleep and do what exercise you can. Be creative to fill your time and support others. Mindfulness and breathing exercises can help reduce anxiety. Phone for professional help

if you become very anxious or depressed. Funerals are very important in traditional societies. Banning or limiting attendance at funerals is necessary, but can be particularly upsetting.

1-28 What are the challenges of locking down poor communities?

While community lockdown is possible in relatively affluent communities it is very difficult in a poor community with many people living together in shanty housing. Income is often on a day-to-day basis so staying at home means no income and no food. Water in homes for washing hands is often not available. The need to get fresh air, sunshine and exercise may be overwhelming. Poor people often have to buy food every day which involves moving around and standing in crowded queues regularly.

With the threat of urban lockdown many people flee to rural areas which only helps spread coronavirus. It remains to be seen whether stopping the spread of COVID-19 is possible in many poor African and Asian countries. If not, the socioeconomic consequences are frightening. Once community lockdown is relaxed there is a real threat that the spread of COVID-19 will return. Therefore, a carefully phased approach to lifting lockdown is needed.

1-29 What is contact tracing?

This is a vitally important part of slowing down the spread of infection. All contacts with the patient in the last few days should be identified, traced and screened for COVID-19. This requires the time and effort of many people but is important in detecting people who are at high risk of incubating or having COVID-19. For countries where transmission in communities is widespread, contact tracing may become impractical. In such cases quarantining all household members

following confirmation of coronavirus infection, is critical in preventing further spread outside of the household.

Clinical features of COVID-19

1-30 How does COVID-19 present?

The infection may not cause any clinical symptoms (asymptomatic) or may present as a mild, moderate or severe illness. The common presenting features are:

- Temperature of 38℃ or more or feeling feverish
- Cough
- Sore throat
- Marked lethargy (tiredness) and general weakness
- Muscle aches (‘flu-like’ symptoms)
- Shortness of breath
- Headache
- Runny or blocked nose
- Abdominal pain, nausea, vomiting and especially diarrhoea
- Loss of smell and taste
- Conjunctivitis (discomfort and redness of the eyes).

1-31 What are the features of mild COVID-19?

Most patients with mild COVID-19 will have a fever at some point during the illness. Other common symptoms are a dry cough or sore throat. A runny nose and sneezing are less common and suggest another cause. In some people the illness is very mild, resembling the common cold.

Patients with a high fever that does not respond to paracetamol or any breathing difficulty have a moderate or severe infection. The clinical condition of those with a mild illness may rapidly deteriorate often with breathlessness and ‘silent hypoxia’.

10-32 What is the most important complication of COVID-19?

Pneumonia. This presents with shortness of breath (difficulty breathing), fast breathing and cyanosis in room air (low oxygen saturation under 92%). Severe pneumonia can progress to acute respiratory distress syndrome, multi-organ failure and death. Hypoxia may be missed clinically if the oxygen saturation is not measured. Patients with a mild illness may suddenly deteriorate.

NOTE

> Acute respiratory distress syndrome occurs when severe viral lung infection causes inflammation with thickened alveolar walls, interstitial oedema and fluid filled alveoli. This reduces gas exchange and increases the work of breathing resulting in hypoxia and respiratory failure. The inflammatory response can become excessive and systemic (cytokine storm) leading to renal and liver failure as well as cardiac or cerebral damage, shock and death.

Management of people with suspected coronavirus infection

1-33 What is the management of asymptomatic people with suspected coronavirus infection?

Well people with suspected coronavirus infection should remain at home and monitor themselves for early symptoms of COVID-19, i.e. they should be isolated at home and self-monitor. They should take their temperature regularly especially if they are feeling unwell. If they develop a fever, cough or breathing difficulty they should phone for medical advice or help, and not

immediately go to their family doctor or the local clinic or hospital where they may infect others or get infected themselves. A healthcare provider will direct them to the correct facility for screening. If they do go to a health facility for assessment or testing, they should inform the staff that they are concerned they may have COVID-19. The staff may ask them to put on a surgical mask and wait in a separate area away from other patients.

The management of people who are well but have been exposed to someone with COVID-19 is the same as those with suspected infection, i.e. quarantine at home and self-monitor. They should have access to accurate information on COVID-19.

If possible, anyone with suspected COVID-19 should be tested for coronavirus infection.

1-34 What should a patient with suspected COVID-19 do when they go to a health facility?

The steps are:

- Wear a cloth mask if they have one, or a bandana, buff or scarf to cover nose and mouth
- Perform respiratory and hand hygiene while awaiting their consultation
- Keep a safe distance from other patients
- Ideally wait in a separate area designated for patients with suspected COVID-19
- Give the details of any contacts so that they can be traced if necessary
- Leave the facility as quickly as possible if they are not admitted to hospital.

1-35 How are patients screened for COVID-19?

The steps are:

- Take a history for typical symptoms

- Record the temperature
- Take a nasopharyngeal or oropharyngeal swab for a screening test to identify the virus.

1-36 Who should be screened?

Each country's national communicable diseases authority will determine the specific criteria for COVID-19 testing, based on international guidelines, availability of laboratories, test kits and the stage of the outbreak. Most screening criteria includes the presence of upper respiratory tract symptoms such as cough or sore throat, shortness of breath or fever plus either:

- History of direct contact within the past 14 days with someone known to be positive for coronavirus infection or suspected to have COVID-19
- History of being in an area known to have high rates of COVID-19 transmission in the past 14 days.

People who have no symptoms but are concerned that they may have been in contact with someone with COVID-19 infection should be self-quarantined at home and monitor themselves daily for development of fever or other symptoms of COVID-19. With a severe shortage of testing kits, strict indications for screening should be applied. Access to more testing kits will allow screening of anyone with respiratory symptoms. Mass screening of whole communities will also become possible. It will also be important for health workers who come into contact with patients, to conduct daily self-monitoring for symptoms of COVID-19.

1-37 How should patients with suspected COVID-19 be triaged?

In order to provide appropriate management fairly and avoid wasting precious resources such as testing kits, hospital spaces and oxygen, all patients suspected of COVID-19 infection (people under investigation) must be triaged on entering the facility into different categories:

- Those where infection has been excluded can go home to maintain social distancing to avoid contracting the disease in future
- Patients with symptoms of mild infection who can isolate and self-monitor at home
- Patients with moderate infection who need admission to an isolation ward in hospital
- Patients with severe infection who need urgent admission to an intensive care unit.

All patients suspected of COVID-19 should be kept in a special waiting area so as not to mix with other patients.

Management of patients with mild COVID-19

1-38 What is the management of patients with mild COVID-19?

The current management is symptomatic and supportive care. Paracetamol (Panadol) is best to reduce fever. Anti-inflammatories such as ibuprofen (Brufen) should be avoided as they may make the condition worse. Antibiotics will not kill the coronavirus and should not be used, unless prescribed by a doctor for co-existing bacterial pneumonia. Keep well hydrated.

Most infected people will have a mild illness and can be managed at home provided they self-isolate and self-monitor. They should have access to correct information on COVID-19. The symptoms usually improve after a few days and disappear by 14 days. Home management reduces the pressure of hospital beds for more severe cases.

1-39 How should patients with mild COVID-19 self-monitor?

With conservative management at home it is important that patients self-monitor to detect any deterioration early. If the symptoms get worse or the fever does not respond to paracetamol the person should contact their healthcare provider, such as a family physicians, clinic or hospital. Patients on home management must have a contact number that they can phone if they need help or advice.

It is very important to recognise when the symptoms get worse as these patients may need to be admitted to hospital for further monitoring and treatment. Most patients requiring hospital care are admitted to hospital on about day 7 of their illness. Shortness of breath is an important symptom as it indicates lower airways disease which may need oxygen therapy.

1-40 Can patients with COVID-19 still infect others after their symptoms disappear?

Some patients continue to shed the virus after their symptoms have disappeared. It is recommended that patients with mild COVID-19 self-isolate for 10 days after the onset of symptoms. If they are asymptomatic but screen positive for COVID-19 they should also self-isolate for 10 days. Coronavirus is most infectious in the first few days after infection and is no longer infectious after 10 days.

Management of patients with moderate or severe COVID-19

1-41 What is the hospital management of patients with moderate or severe COVID-19?

Some patients start with the symptoms of a mild infection but during the first few days these progressively worsen with a high temperature. They have difficulty breathing and may become cyanosed.

All patients with moderate or severe COVID-19 must be admitted to hospital. Their management will be determined by the hospital management protocols and available equipment. It is important that patients wear a surgical mask to reduce the risk of droplet spread to others. The patient must be isolated in a specially designated COVID-19 area.

Patients with moderate illness do not need admission to an intensive care unit and most can be managed in an isolation ward and discharged home within 1 to 2 weeks. Observing vital signs and the oxygen saturation is important. Other than temperature control, oxygen therapy and general support there is no specific treatment for the viral infection as yet (consult up to date international and national guidelines as treatment protocols are regularly updated). Detailed management should follow hospital protocols. Close monitoring for hypoxia with an oxygen saturation below 94% is essential.

Patients with severe illness get progressively worse and most need ICU care and ventilatory support. Admission to an ICU is usually about day 10 of the illness. The outcome of most critically-ill patients requiring ICU support for COVID-19 disease has been poor, with low survival rates.

Intravenous dexamethasone, immunoglobulin, CPAP or high flow nasal oxygen for severe illness reduces the need for intensive care and ventilation. If the availability of ICU facilities and ventilators is overwhelmed by the number of patients a system of triage will be needed. In this tragic circumstance a clearly understood and agreed upon protocol of who gets admitted for intensive care must be developed. A frailty assessment scale may be helpful. Plans for compassionate palliative care of the dying, often without their relatives, is essential.

Protecting healthcare workers

1-42 What can healthcare workers do to protect themselves?

They should:

- Adhere to handwashing, respiratory hygiene and social distancing whenever possible both at home and at work
- Ask all patients with respiratory symptoms to wear a surgical mask when near other patients or healthcare workers
- Adhere to all hospital or clinic protocols
- Use personal protective equipment when appropriate
- Not touch their face with their hands or gloves
- Support each other during this stressful time.

1-43 What is personal protective equipment (PPE)?

Personal protective equipment (PPE) is the equipment needed to protect healthcare workers from becoming infected with coronavirus. It is essential that healthcare workers protect themselves when managing patients with COVID-19. The willingness of healthcare workers to manage these patients may be dependent on the availability of PPE. The most appropriate and effective PPE should be used in each circumstance. Do not use PPE unless it is indicated. Basic personal protective equipment (PPE) consists of:

- A plastic apron or a non-sterile long-sleeved gown
- Non-sterile gloves

- Surgical masks for most staff and a well-fitted N95 respirator for clinical staff while performing aerosol-generating procedures
- Eye protection such as goggles or a face shield.

Donning (putting on) and doffing (taking off) full PPE is a skill that needs practice and must be done correctly to avoid healthcare workers being contaminated with coronavirus. View this demonstration videos for safely putting on and taking off PPE.

Doning PPE: https://www.youtube.com/watch?v=H4jQUBAlBrI

Doffing PPE: https://www.youtube.com/watch?v=PQxOc13DxvQ

1-44 Can personal protective equipment be reused?

Before considering reuse, first ensure that a restriction policy is in place, to make sure PPE is only used by those who need it i.e. healthcare workers in close contact with patients with suspected or confirmed COVID-19. Secondly, consider extended use, that is wearing the PPE for longer that would usually be the case (e.g. wearing a surgical face mask for the entire shift). The last option is reuse of PPE, when supplies are very limited. Reuse of PPE (PPE conservation) is still preferable to having no PPE and remains a major challenge all over the world. Aprons and gloves should be replaced between handling patients. Goggles and face shields can be washed clean and disinfected. Surgical masks can be used for 8 hours during a work shift but should be discarded if they become moist or get soiled, or at the end of the shift.

1-45 For which procedures are N95 respirators needed?

N95 respirators are only needed by clinical staff when there is a risk of aerosolizing (airborne) coronavirus, i.e. during aerosol generating procedures such as the collection of nasopharyngeal and oropharyngeal swabs, tracheal aspirates, or bronchoalveolar lavage for coronavirus testing, manual bag-mask ventilation, non-invasive CPAP ventilation, tracheal intubation, open suctioning, tracheotomy, bronchoscopy, endoscopy, ENT procedures, dental procedures, maxillo-facial procedures and cardiopulmonary resuscitation. A number of different N95 respirators designs are available e.g. cone/cup, duckbill and fold-flat.

For the general care of patients with respiratory symptoms, surgical masks can be used to protect the healthcare worker's mouth and nose from respiratory droplets. Staff are often asked to use the same surgical mask over the shift.

1-46 Can one reuse a N95 respirator?

Yes. If a N95 respirator (special face mask) is indicated for aerosol-generating procedures when you are working in a COVID-19 ICU or triage area, use the same respirator for your entire shift. For reuse, carefully remove the N95 respirator using a clean paper towel and store the respirator in a clean paper bag, labelled with the staff member's name. Do not crush or crumple it. Ensure it still fits securely around the face and nose when you use it again by performing a seal check: when breathing in or out suddenly the respirator should collapse or expand slightly without any leak of air. A N95 respirator can be reused so long as it is not damaged, a seal test is normal and the respirator is not damp or crushed. As N95 respirators are often in short supply they often need to be used repeatedly.

1-47 How should you handle your uniform or work clothes?

Ideally, used scrubs are laundered at the facility. If this is not possible, remove your work clothes at the workplace and place them in a plastic bag. If removed at home, place them in a washing basket immediately. Wash your hands after removing your clothes. Wash the clothes in hot water if possible (60°C). Clean any spectacles with disinfectant when leaving the COVID-19 ward. Ensure your cell phone has been cleaned.

1-48 How should infectious waste be handled and disposed of?

The usual procedures for the collection and disposal of infectious waste in red bags applies. No additional precautions are needed.

1-49 How should contaminated linen be handled?

The usual procedures for the packaging and sealing of potentially contaminated linen is the use of alginate (special polythene) bags or, where not available, clear plastic bags.

1-50 What cleaning of surfaces and equipment is required in COVID-19 areas?

Increased frequency of environmental cleaning of healthcare facilities is very important. This will reduce the chance of spreading coronavirus from contaminated surfaces and equipment. Surfaces and equipment should be cleaned at least twice daily with detergent (soap and water). Focus cleaning on high-touch surfaces, e.g. bed handles, tables and phones. In COVID-19 wards, aim for more frequent detergent cleaning, followed by disinfection with a 0.1% concentration of sodium hypochlorite (add 2 ml bleach to 1 litre of water). Disinfect the equipment with 70% isopropyl alcohol after each use.

1-51 What protection should community health workers use?

Community health workers handing out education material to the general public should maintain a safe personal distance of at least 1 metre but do not need masks or PPE. Although surgical masks or PPE are not needed for outdoor contact, they should wear a cloth mask when in public.

1-52 What is a screening and testing triage area outside a hospital?

Some hospitals have set up a triage area where possible patients can be screened for symptoms of COVID-19 or recent contact with a COVID-19 infected person. This prevents people with coronavirus infection entering the hospital when they could be managed with self-isolation at home. Natural ventilation and adequate light is needed. Often a temporary tent is used for a triage facility. While awaiting triage people must not be crowded but kept at least 1 metre apart and practice respiratory hygiene. They should wear face covering or cloth masks.

1-53 How can coronavirus spread be limited in hospitals?

There are a number of steps that can be taken in hospitals and other health facilities:

- Only admit patients if the indications for hospital admission are present
- Patients with respiratory symptoms must be triaged into a separate area and wear a surgical mask
- All patients suspected of COVID-19 must be urgently tested
- Patients with suspected COVID-19 must be separated from other patients in a dedicated (cohort) area
- Patient with proven coronavirus infection must be cared for in dedicated COVID-19 wards

- Limit the number of entrances to control movement into and out of the hospital
- Restrict or reduce the number of visitors allowed into the facility
- All in-patients should be screened daily for COVID-19 symptoms
- Avoid moving or transferring patients
- Keep beds as far apart as possible to reduce droplet spread
- Increase frequency of surface and equipment cleaning
- All staff and patients must adhere to physical distancing and hand hygiene, e.g in tea rooms and staff meetings
- Staff in contact with COVID-19 patients must wear a gown, surgical mask, eye protection and gloves
- Adequate PPE must be available at all times
- All staff should have training in COVID-19 prevention and perform daily self-monitoring for COVID-19 symptoms. Influenza immunisation should be encouraged.
- All staff must wear non-medical cloth masks on public transport
- Allow all staff access to testing if they are concerned that they may be infected.

Case study 1

A man aged 72 years with chronic lung disease due to many years of smoking cigarettes has recently returned from a business trip to USA. Two days after getting back he develops a dry cough and fever. He visits his family physician who is concerned that he may have COVID-19.

1. Why should the doctor worry that he may have COVID-19?

Because he has recently visited an area which has a COVID-19 epidemic and he has symptoms that suggest COVID-19.

2. Does he have any factors which place him at high risk of severe infection?

Yes. Both his age and his chronic lung disease due to smoking are high risk factors.

3. Should he have gone to the doctor?

No. He should have phoned his doctor for advice. While sitting in the waiting room he may have infected other patients and the staff.

4. How could the diagnosis of COVID-19 be confirmed?

By taking a nasopharyngeal or oropharyngeal swab and sending it for a coronavirus screening test. A positive test would confirm the clinical diagnosis.

5. How severe is this patient’s illness?

At present it is mild but it may become worse over the next few days. Careful self-monitoring is therefore important.

6. How should he be managed?

At this stage he can be isolated and managed at home. Paracetamol can be taken for fever and body aches. He should report to his doctor if his symptoms get worse. With a mild infection he should be feeling better after about a week. Every effort must be taken to prevent the spread of infection to others and his contacts should be traced and screened for infection.

Case study 2

A worried woman contacts her local clinic for advice as her best friend, who she meets daily for tea and chit chat, has been diagnosed with COVID-19. She has been watching TV programmes on

COVID-19 and knows the importance of self-isolating if she might be in the incubation period. She feels anxious and has a headache.

1. How long will she need to wait if she is to develop symptoms of COVID-19?

The average time period between exposure to the coronavirus and the onset of symptoms of COVID-19 is 5 days but it may be as long as 14 days.

2. Is it safe for her 10-year-old daughter to visit her if they do not touch or kiss?

No. Self-isolation means no contact with other people. If she has early, mild symptoms such as a headache she may be infectious. She should self-isolate and self-monitor for 10 days after her last contact with the COVID-19 infected person.

3. If she went to a coronavirus screening centre should she wear a mask?

If she has a cloth mask this will reduce the risk of her spreading the coronavirus by coughing or sneezing. However a cloth mask worn by others is unlikely to prevent them getting infected. Social distancing and frequent handwashing are the best ways the general public can protect themselves.

4. When will she be feeling better if she has mild COVID-19?

With a mild COVID-19 infection she should be feeling better in 7 to 14 days. She should remain at home until she is fully recovered. If her symptoms become worse or she gets short of breath she should immediately seek advice as she may need to be hospitalised.

5. Would influenza immunisation have protected her from COVID-19?

No. However being immunised against influenza is recommended as ‘flu’ can lower one’s immunity to other infections. Being immunised also makes it more likely that any flu-like symptoms are due to COVID-19.

Case study 3

A district head quarter (DHQ) hospital medical superintendent is setting up a ward for patients with moderate COVID-19. He is discussing the requirements to protect his staff, especially the nurses who will be caring for the patients. They have expressed concern that they will become infected and then pass the infection on to their families. He reassures them that they will have personal protective equipment.

1. What is personal protective equipment?

PPE consists of a mask, plastic apron or non-sterile long gown, non-sterile gloves and eye protection.

2. Is a surgical mask adequate?

Usually it is. However a N95 respirator should be worn for procedures such as taking an oropharyngeal sample, where the virus may pass into the air.

3. What is a N95 respirator?

It is a special face mask that prevents virus in the air being inhaled. However the N95 respirator must be tight fitting without air leaks.

4. Can a N95 respirator be reused?

Due to shortage of equipment N95 respirators may need to be reused. This can be done safely provided they are not damaged, there is no air leak and they are dry.

5. What should be used to disinfect hospital equipment?

After each use equipment should be disinfected with 70% isopropyl alcohol.

6. What advice should be given about removing work clothes?

This is best done in the workplace. If the clothes cannot be washed at the facility they should be taken home in a plastic packet. Wash in hot water. Do not forget to wash your hands after touching the clothes before they are washed.

Adapted from

1. Book - Infection Prevention and Control Paperback – August 23, 2020 edition by Dr Angela Dramowski (Author), Prof David Woods (Author), Prof Shaheen Mehtar (Author)

2. World Health Organization (WHO): www.who.int

3. Centers for Disease Control and Prevention (CDC): www.cdc.gov
