# Supplementary material for: WhatsApp-Delivered Intervention for Continued Learning for Nurses in Pakistan During the COVID-19 Pandemic: Results of a Randomized-Controlled Trial
Source: Front Public Health. 2022 Feb 15;10:739761. doi: 10.3389/fpubh.2022.739761 (PMC8885589; doi:10.3389/fpubh.2022.739761)
Supplement: Supplementary file 3 [file Data_Sheet_3.pdf]

<u>Intervention Training Material for Nurse Leadership Study</u>

Module 3: Nurse leadership in Infection Prevention and Control

Contents

Intervention Training Material for Nurse Leadership Study.................................1

1. Objectives 2
2. Defining Leadership in IPC 2
3. IPC focal person key roles and tasks 3
4. Characteristics of a leader..5
5. Types of leadership 6
6. Components and channels of communication 8
7. Managing conflict 10
8. Handling Conflict..11
9. Types of conflict 14
10. Confronting conflict 15

1. ***Objectives***

- Define leadership as it applies to the roles and responsibilities of the IPC focal person.
- Identify how characteristics and types of leadership relate to your own leadership approaches.
- Define the components of communication and describe how they are used to communicate effectively in IPC.
- Select the most effective channels of communication to use in various IPC situations.
- Explain which leadership skills and behaviours are needed for optimal conflict resolution.

2. ***Defining Leadership in IPC***

While there are many definitions of leaders and leadership in different fields, the following definition is particularly fitting in relation to IPC leadership.

'Leadership describes the ability to influence, motivate, and enable members of an organization to contribute to the effectiveness and success of the organization'[1]

This definition emphasizes the 'soft' skills a leader needs as well as focuses on the members of a given organisation.

An IPC leader should seek to continuously improve implementation of IPC programmes and all core components. As a leader you will use those soft skills to influence multimodal strategies to:

Build a system (including infrastructures) that supports IPC practices.

Teach others about IPC.

Check that your organization is doing the right thing at the right time.

Sell it to others and keep them interested in adopting and maintaining excellent IPC practices.

Live it by embedding excellent IPC practices across your healthcare facility's culture.

Effective IPC leadership has an impact! Leaders in close and regular contact with clinical teams in wards and units positively influence quality of care. Leaders support others to develop, implement, and evaluate their own solutions to problems. For instance, leadership associated with hand hygiene, gowning, and gloving has influenced greater quality of sanitation in many facilities.[2] Leadership that promotes IPC practices are associated with reduced incidence of pneumonia and urinary tract infections.[3] Remember, using leadership skills to engage staff and influence hospital managers can improve safety and quality of care in your facility.

3. *IPC focal person key roles and tasks*

The IPC focal person will need to apply leadership skills to a variety of situations. Let's look at the key roles and tasks that will require those skills. The IPC focal person is a builder and nurturer. It is your duty, as the IPC focal person, to develop and sustain many coordinated activities that promote safety and quality of care for staff and patients. The IPC person is a builder and nurturer.

As a Builder:

- Develop, implement, coordinate, and evaluate the IPC programme.
- Develop and support implementation of IPC activities at facility or district levels.
- Develop, update, and manage IPC strategies, guidelines, and all tools and resources.
- Develop surveillance systems for HAI, etc. in collaboration with epidemiologists and surveillance team.

As a Nurturer:

- Liaise with relevant hospital/district departments to ensure integration of IPC activities.
- Audit and monitor progress of facility IPC plan.
- Interpret and communicate data on infrastructure, process, and practice indicators for decision-makers.
- Sustain IPC workforce through training.
- Raise awareness of HAI and AMR among the public and health care professionals.
- Offer advice about IPC supplies, technical specifications, and procurement systems.

A wide variety of skills, domains, and tasks are needed for implementing IPC; this means that you will need to collaborate, influence, and work towards integration with a number of other programmes/services, perhaps even outside the health sphere. This graphic illustrates the most important programmes/services that may tie in with IPC. Can you identify some additional relevant programmes that you may need to collaborate with when implementing IPC policies in your local context?

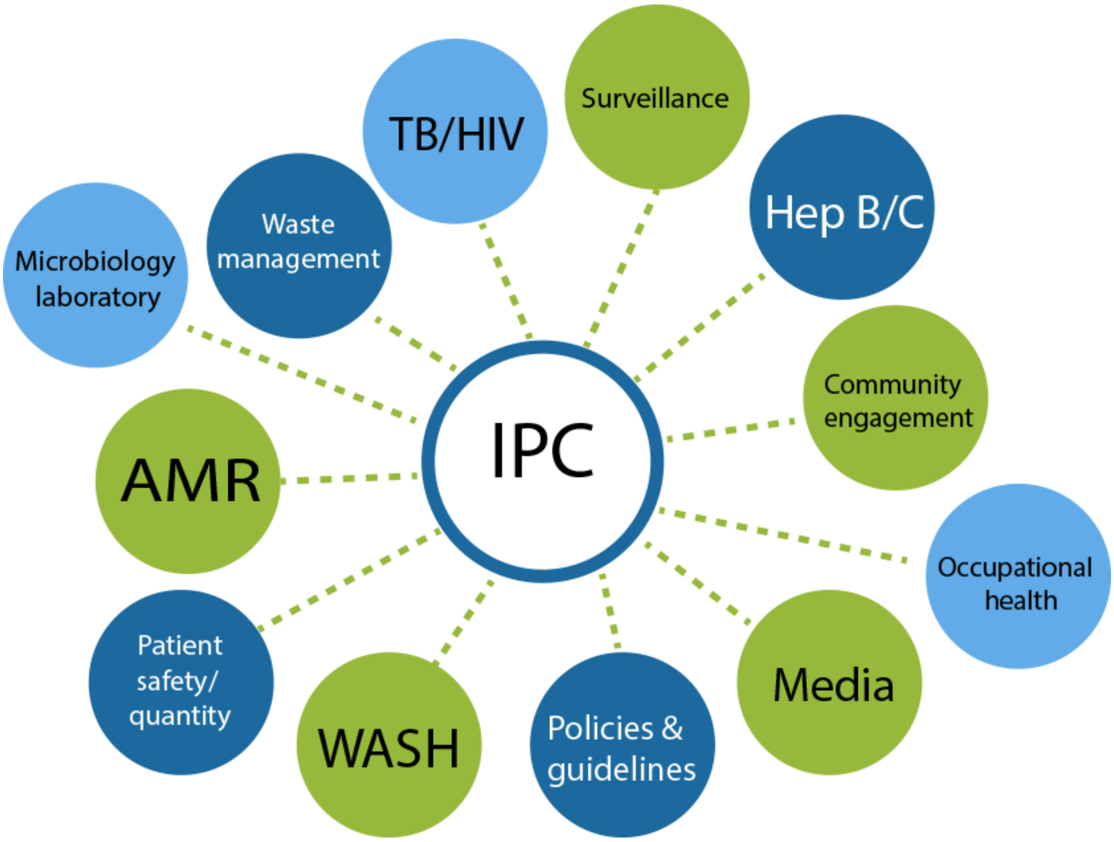

(Source: Public file-

https://depts.washington.edu/edgh/app-ipc/web/leadership_skills.html)

4. Characteristics of a leader

A leader can be someone who is strong, inspirational, decisive, and unique in an organization. They may observe from a distance, only getting involved in key moments. They may be seen as superior to other members of the organization. They may be seen as a visionary person who can convey a culture of excellence that others aspire to.

A leader may be more like a peer within a given organization - they may carry out a specialized role. They may be, and often are, replaced by other people. This type of leader is specifically focused on the task or outcome rather than their position.

A leader may simply be someone who asks for help as much as they provide help. They are able to connect people and assets within an organization. These leaders are likely more focused on developing strategies based on resource availability.

Leaders are often identified by their personality, their behaviour, and their actions. They are defined by how they deal with themselves, with others, and with the projects they lead.

For IPC the characteristics of a leader expand to include more specific traits. As an IPC leader you are expected to foster a culture of excellence, develop and communicate a vision about the organization, and anticipate potential problems.[4] While these ideas may be applicable to leaders in any field, it is important to remember that ideas or perceptions of leaders and leadership will be shaped by the overall culture and norms of the society.

5. *Types of leadership*

The characteristics of a leader also relate to their leadership style. Just as every leader is different, the type of leaders you will encounter or become will be different.

Situational Leadership: Situational leaders adapt their leadership style to individual situations. This type of leadership is based on a relationship between the leader's supportive and directive behaviour and the follower's levels of development.[5]This leadership style requires personal involvement, sustained communication, and emotional support. Situational leaders are fluid and able to adapt their leadership to a given situation, to the resources available, and the strengths and weaknesses of the team. This flexibility demands a solid relationship between leaders and followers. These leaders are willing, and interested, in investing time and energy communicating and providing support. In situational leadership the context shapes how the leader

behaves. They may adapt their style to the context they find themselves in. e.g Quaid e Azam

Transformational Leadership: Transformational leaders are less relational and emotional in their interactions with followers. They thrive on developing and communicating a vision and empowering followers to embrace that vision. Transformational leaders motivate their followers by appealing to their ideals, empowering and inspiring them to use their own beliefs and personal strengths. These leaders have the ability to inspire change in their followers so that they become more effective and engaged. They have and share a vision for what an organisation should and could be. This type of leader develops others to exceed their own self-interests for a higher purpose.[6]The leader-follower relationship is based on interactions or exchanges.[7] Famous transformational leaders are thought to typically be religious leaders. However, we also find transformational nurse and doctor leaders. You too could develop into a transformational leader! E.g Nelson Mandela and Mother Teresa

Transactional Leadership: Transactional leaders are more focused on the analytical aspects of their role. This includes evaluation, measurement, and standardization aligned with performance. They are focused on the evaluation, training, and correction of subordinates. They are often driven by mandates from higher leaders. The transactional leader values hierarchy, and may be a visible leader at the top of the chain. These leaders function within a clear chain of command, motivating their followers through reward and punishment. Often followers who fail to live up to their requirements are punished. For transactional leaders, their key motivation is to be effective and efficient.[8] e.g Bill Gates

After learning about these broad types of leadership, in a notebook, or with a partner, discuss what type of leadership best fits your personality and values. Which leadership

approaches do you feel will best help you to manage a successful IPC programme? Who are some leaders you admire? Which type of leadership does that leader embody?

6. *Components and channels of communication*

Effective communication is another critical skill an IPC leader needs. Many situations in IPC require effective interpersonal communication. Whether you are implementing a new way of doing things, dealing with infection outbreaks, providing feedback of an IPC assessment, convincing senior managers about the importance of investing in IPC, or aiming at modifying behaviors of health professionals and patients, effective communication is required for a successful IPC programme or project.

Communication involves thoughts and feelings, either explicit or implicit.[9] These emotions occur with (explicit) or without (implicit) the person's conscious awareness. This includes non-verbal communication. Good communication allows people to speak and be listened to, ask questions, and express thoughts without interruption in an understandable manner.

Seven key elements that are essential in the process of communicating information:

The Players: The people involved may include the recipients of an innovation you would like to implement or the targets of a behavior you would like to change, administrators who may help support the implementation of your idea, your staff, and even the public.

The Exchange: Make sure that the messages you communicate are clear and concise. Different audiences may perceive or interpret messages in different ways. Keep that in mind as you choose the most effective channels and methods of communication.

The Channel: Selecting the right channel for a given audience will be the most effective way to communicate. You may choose to communicate the same message to

different groups via different channels. The following is a non-exhaustive list of communication channels that you might use:

- In person meetings (face to face)
- Formal education (university)
- Informal training (bedside training)
- SMS
- Mass media such as television and radio
- Telephone
- Meetings
- Policies and guidelines
- Enforcing regulations
- Care pathways (written directions on process of care)
- Information packets or handbooks
- E-learning systems
- E-mail
- Social networks and websites
- Banners or posters

Some other effective of ways to communicate within an IPC programme are:

- Develop leaflets for patients and family members or staff.
- Lead multidisciplinary teams during outbreak investigations.
- Report to hospital management on performance indicators.
- Respond to journalists about hospital performance or an event (an outbreak).
- Present a successful hand hygiene programme at a conference.
- Advocate for more resources (including an IPC budget).

The Noise: Noise can refer to unnecessary words, pictures, sounds, or graphics that don't support your message. Keep in mind that the most effective messages are clear, easy to understand, and concise.

The Context: In addition to choosing how your message is communicated, you must focus on other contextual factors. This may include ***when, how often, to whom, and for how long*** you should communicate your message.

The Feedback: A good way to determine if your messaging has been effective is to ask for feedback. You can supply a phone number, email address, survey, or comment card to collect information from recipients.

The Impact: Overall, analyze the impact your messaging has made on the community you intended to influence. Feedback is a good way to collect this data, as well as observation and conducting surveys.

7. *Managing conflict*

Another important skill for IPC leaders is knowing how to manage conflicts. When introducing a systemic change, such as introducing a new guideline, conflicts may arise. For example, introducing a new wound dressing technique could threaten the status quo. People who were comfortable with the "old routine" may feel uncomfortable having to learn a new approach, and this may result in challenges from different members of the team.

Conflict and tension naturally occur among co-workers and within organizations. According to Barki & Hartwick, conflict is "A dynamic process between individuals and/or groups as they experience negative emotional reactions to perceived disagreements and interference with the attainment of goals."[10] Sometimes facilities or national programs need to quickly implement new IPC practices, which can lead to

conflict among the IPC team (about how to make these changes), among staff at the facility, and among stakeholders. Conflict in itself is not necessarily a negative thing. Conflict can be used to address or critique shortcomings of proposed interventions and their implementation. Do not ignore the psychological effects of sudden, drastic, or dramatic change. Individuals may have feelings of fear or insecurity about these interventions. Each of the Core Components is complex in the sense that it requires several independent actions, behaviours, and attitudes to come together. This can be stressful and demand excellent communication skills.

As an example, before finalizing and disseminating new guidelines, stakeholders should be consulted so that they can give feedback about how these guidelines would affect their positions. Here's another example: Successful implementation of new surveillance or monitoring and evaluation systems is likely to require input from several departments. Goals, processes, rationales, etc. must be clearly communicated to staff in these departments or else there could be negative conflict. When anticipating conflict, consider interactions between team members and the environment, how you should respond, and which organisational supports may aid in resolving those conflicts.

8. *Handling Conflict*

Think about your leadership style. How might your leadership style affect the way that you address and resolve conflict? You may want to examine whether your approach tends to focus on the benefit to you (assertiveness) or the benefit to everyone (cooperativeness).

Based on the different combinations of assertiveness and cooperativeness, Ken Thomas and Ralph Kilmann identified five modes of responding to conflict. They developed the **Thomas-Kilmann Conflict Mode Instrument**, which is an assessment

used around the world to handle conflict. This graphic, adapted from Kilmann Diagnostics.

https://www.youtube.com/watch?v=PFIydyH2H8Y

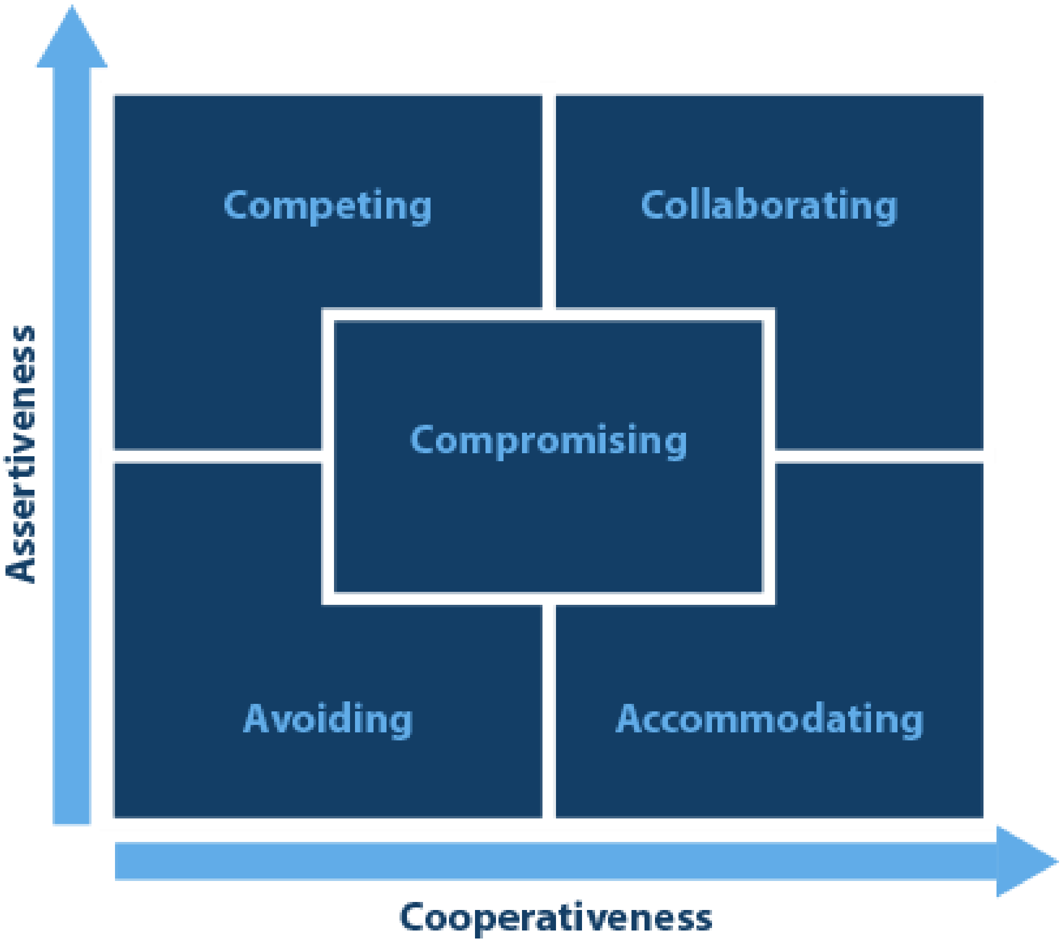

(Source: Schaubhut, N. A. (2007). Thomas-Kilmann conflict mode instrument. CPP Research Department)

Compromising: As a strategy, compromise can best be described as "meeting in the middle". Together, both parties work to find a mutually acceptable decision. In many cases, neither party gets exactly what they want. This is okay, because the most important thing is that they have reached a comparable solution together. This approach is somewhat assertive, somewhat cooperative. While compromising is a

good middle of the road option, keep in mind more creative solutions may be neglected for both parties to agree on a solution.

Competing: When a difficult situation calls for a quick decision, competing is an effective approach. If there is no gray area in this situation, or time is the most pressing factor, you may need to assert power to achieve the best outcome. This can be an unpopular, but necessary way to handle a problem. You may feel that you are "throwing your weight around", and others may perceive your actions negatively. Be careful when using this approach, as it may alienate others involved. Determine whether competing will resolve the problem, or make it worse.

Collaborating: A collaborative approach to conflict resolution means that both parties agree to a new solution. This means that the parties involved must be willing to engage to find a solution that works for everyone. This approach is considered to be the most enduring and productive to find a solution, but it involves the most energy and emotional intelligence. This means an ability to recognize and manage the emotions of oneself and others. This highly assertive, highly cooperative approach is best used when parties involved are comfortable with ambiguity. That is, they are able to see the gray area in a situation.

Avoiding: Avoiding is an approach to consider when a confrontation may cause more harm than good. Some conflicts resolve themselves. Choosing not to engage directly may allow you to take a step back to better assess the situation. In some situations it is useful to buy yourself some time and not react too quickly. You may delegate the task of resolving the conflict to another member of the team as an exercise in team building or a lesson in leadership. However, this may be seen as avoiding responsibility, or being unproductive. It is your decision to assess whether direct action is necessary before the problem gets worse.

Accommodating: An accommodating approach to conflict is useful when the relationship between the people involved outweighs the situation at hand. In other words, it may be more important to give up power in a situation rather than harm a good working relationship. You may choose to be accommodating when maintaining peace or creating goodwill is the more important outcome. To do this, you must be willing to give up your concerns or interests in the situation. This approach, while very cooperative, is unassertive. It may be productive in the moment, but may prevent the people involved from finding a more creative solution to the problem.

9. *Types of conflict*

As a leader you will have to manage and resolve conflicts in order to move forward productively. It is important to first identify the type of conflict to be able to approach the problem appropriately.

There are two types of conflict: task-related and relationship-related. Task-related conflict can produce creative results to difficult problems when handled correctly. Relationship-related conflict can affect the way members of a team function together and hinder the progress of your goals.

When addressing conflicts, keep perspective in mind. Your perspective, history, and context of the situation may be different than the other people involved. Be sure that everyone involved has had the opportunity to be heard.

Task-related Conflict: Task-related conflict can arise by nature of a simple difference of opinion of how a job can be done. This type of conflict can be productive. When passions are high and people are engaged, many creative ideas can be introduced. Handled correctly, you can produce results that will strengthen your team or activity. However, if the conflict goes on too long, or is not resolved, it could damage the way that your team functions. To overcome this type of conflict, refer to

the "cooperative" modes of conflict resolution. Think of accommodation, collaboration, and cooperation when you consider the ways in which you will choose to engage with the problem. For task-related conflict, work together to find solutions that address the needs of the people involved.

Relationship-related Conflict: At times, task-related conflict can evolve into relationship-related conflict. This can happen if the previous conflict was not resolved or not addressed in a timely manner. This can result in misunderstandings, hurt feelings, blame, and miscommunication. Sometimes this type of conflict can arise from personal issues, resentment, or competition. Unlike task-related conflict, relationship-related conflict has no productive outcome. This type of conflict is very personal, and you may not be able to resolve it completely. Your goal should be containing the conflict and keeping it from getting destructive.

10. Confronting conflict

In the following Everyday Leadership video Confronting Conflict, Tachi Yamada encourages you to face conflict head on. This video shares best practices when confronting conflict before problems get worse.

(Video clip is attached in folder)

Adapted from: https://depts.washington.edu/edgh/app-ipc/web/leadership_skills.html

References:

1. Bass BM (2008) The Bass Handbook of Leadership: Theory, Research, and Managerial Applications. Fourth edition. Free Press, New York NY.Health Serv Manage Res. 2007 Aug;20(3):183-8.

2. Weiss, S. A., Tappen, R. M., & Grimley, K.,. (2010). Essentials of Nursing Leadership and Management: F. A. Davis Company, Philadelphia.

3. Johansen, M. L. (2012). Keeping the peace: Conflict management strategies for nurse managers. Nursing Management, 43(2), 50-54.
