# Supplementary material for: WhatsApp-Delivered Intervention for Continued Learning for Nurses in Pakistan During the COVID-19 Pandemic: Results of a Randomized-Controlled Trial
Source: Front Public Health. 2022 Feb 15;10:739761. doi: 10.3389/fpubh.2022.739761 (PMC8885589; doi:10.3389/fpubh.2022.739761)
Supplement: Supplementary file 4 [file Table_1.pdf]

Supplementary Table 1

Demographic characteristics associated with infection control, coronavirus knowledge, and leadership and conflict management (at baseline)

	IC			CK			L&CM		
	Mean ± SD	Standard Error of Mean	*p* value*	Mean ± SD	Standard Error of Mean	*p* value*	Mean ± SD	Standard Error of Mean	*p* value*
Age<br>20-29 years<br>30-39 years<br>40+ years	 31.55 ± 0.96<br>31.68 ± 0.85<br>30.50 ± 2.50	 0.06<br>0.12<br>1.02	 **0.026**	 31.52 ± 1.13<br>31.59 ± 1.16<br>30.00 ± 3.34	 0.08<br>0.17<br>1.36	 **0.011**	 31.49 ± 1.16<br>31.65 ± 0.80<br>30.83 ± 2.04	 0.08<br>0.12<br>0.83	 0.230
Gender<br>Female<br>Male	 31.55 ±1.03<br>31.54 ±0.98	 1.03<br>0.98	 0.909	 31.52 ± 1.22<br>31.45 ± 1.28	 0.10<br>0.13	 0.688	 31.47 ± 1.16<br>31.55 ± 1.08	 0.09<br>0.11	 0.608
Type of Contract<br>Permanent<br>Temporary	 31.56 ± 0.97<br>31.52 ± 1.07	 0.07<br>0.11	 0.800	 31.57 ± 1.21<br>31.36 ± 1.30	 0.09<br>0.14	 0.222	 31.57 ± 1.04<br>31.37 ± 1.27	 0.08<br>0.13	 0.190
Years of Service<br>> 6 years<br>0-5 years	 31.60 ± 1.00<br>31.42 ± 1.03	 0.07<br>0.12	 0.230	 31.55 ± 1.19<br>31.34 ± 1.37	 0.09<br>0.16	 0.255	 31.53 ± 1.12<br>31.42 ± 1.15	 0.08<br>0.14	 0.492
Monthly Income<br>> PKR 59,000 / USD 383.7<br>< PKR 59,000/ USD 383.7	 31.66 ± 0.77<br>31.50 ± 1.09	 .09<br>.08	 0.276	 31.73 ± 0.84<br>31.40 ± 1.37	 0.10<br>0.10	 **0.049**	 31.59 ± 1.03<br>31.47 ± 1.17	 0.12<br>1.17	 0.451
Last Degree<br>BSc or above<br>Diploma	 31.64 ± 0.88<br>31.46 ± 1.10	 0.08<br>0.09	 0.170	 31.68 0.97<br>31.33 1.42	 0.09<br>0.12	 **0.032**	 31.56 1.10<br>31.45 1.16	 0.10<br>0.10	 0.439
Marital Status<br>Single<br>Currently Married	 31.52 ± 1.02<br>31.60 ± 0.98	 0.08<br>0.11	 0.591	 31.44 ± 1.27<br>31.60 ± 1.19	 0.09<br>0.13	 0.369	 31.45 1.20<br>31.61 0.95	 0.09<br>0.10	 0.302

* Statistically significant relationship ($p < 0.05$) for ANOVA results
